# A comparison of oral microbiome composition between highly trained competitive athletes and untrained controls

**DOI:** 10.1038/s41598-025-16835-3

**Published:** 2025-08-28

**Authors:** Annabel Simpson, Bob T. Rosier, Javier Pons Tamarit, Fiona Henriquez, Alex Mira, Chris Easton, Mia Burleigh

**Affiliations:** 1 Sport and Physical Activity Research Institute, University of the West of Scotland, Blantyre, Scotland; 2 Department of Health and Genomics, Centre for Advanced Research in Public Health, FISABIO Foundation, Valencia, Spain; 3 Department of Civil and Environmental Engineering, University of Strathclyde, Glasgow, Scotland; 4 Institute of Life and Earth Sciences, Heriot-Watt University, Edinburgh, UK; 5 School of Health and Life Sciences, Hamilton International Technology Park, University of the West of Scotland, Stephenson Pl, Blantyre, Glasgow, G72 0LH UK

**Keywords:** Oral microbiome, 16 s rRNA, Exercise, Nitrate-reduction, Nitric oxide, Dental disease, Physical activity, Nitrite-production, Nitrate, Exercise training, Microbiome, Dental diseases, Oral diseases

## Abstract

The oral microbiome has a crucial role in nitric oxide (NO) production and contributes to oral and systemic health. This study compared oral microbiome composition and markers of NO production between highlytrained competitive athletes and inactive controls. Competitive athletes and untrained controls (N = 10 per group) were recruited. Saliva, plasma, supragingival plaque and the tongue dorsum microbiome were sampled. The microbiome was examined using long-read 16S rRNA sequencing and ozone-based chemiluminescence used to measure nitrate (NO_3_^-^) and nitrite (NO_2_^-^) levels. Weekly training duration was recorded and aerobic fitness capacity (V̇O_2max_) assessed via maximal exercise testing.The beta-diversity of the tongue dorsum microbiome differed between groups (Adonis *p* = 0.046) and athletes had a higher relative abundance of NO_3_^-^-reducing *Rothia mucilaginosa* and unclassified *Gemella* species. No significant differences were detected in the supragingival plaque. Positive correlations were detected between *R. mucilaginosa* and unclassified *Gemella* species and aerobic fitness. Athletes had higher levels of salivary NO_3_^-^ (*p* = 0.003) and NO_2_^-^ (*p* = 0.03). Exercise training may impact the tongue dorsum microbiome more than supragingival plaque, with the relative abundance of specific health-associated bacteria higher in the tongue dorsum microbiome of athletes. The robust methodologies employed in this study highlight a possible link between consistent exercise and the development of an oral microbiome conducive to health. However, further research is needed to explore the mechanisms connecting exercise, the oral microbiome, and overall health.

## Introduction

The oral microbiome comprises diverse communities of microbes, including bacteria, fungi, viruses, and protozoa^1,2^, spread across various oral niches, including the teeth, gingiva, tongue, and mucosal surfaces^3^. In each environment, microbial species can contribute to health by preventing the colonisation of foreign pathogens and through symbiotic actions such as metabolism of key nutrients and modulation of the immune response^4–7^. However, if an imbalance occurs, oral microbes can also contribute to the development of oral^8^ and systemic disease^9^.

Individuals who meet minimum recommended levels of physical activity are generally reported to have good oral health^10^. However, it is likely these individuals also perform regular oral hygiene and consume a healthy diet^11,12^. These behaviours reduce the risk of imbalance in the oral microbial ecosystem and subsequent development of dental pathologies, including dental caries and periodontal diseases^13–16^.

In contrast, dental disease prevalence is high among elite-level sportspeople^17^, ^18,19^. The elevated risk has been attributed to lifestyle factors, such as the frequent consumption of simple carbohydrates and suboptimal dental hygiene practices^20,21^, as well as physiological factors related to intense and/or long-duration exercise, including elevated salivary lactate levels^22^,Oliveira et al*.*, 2015) and dehydration^23^. These factors can decrease the salivary flow rate and pH, which may disrupt the oral microbiome^24^,^25^

Exercise training induces other physiological adaptations throughout the body^26,27^, including increased expression of Nitric Oxide Synthase (NOS) enzymes^28,29^, which generate Nitric Oxide (NO) via synthesis of L-Arginine^30^. This has potential benefits for health as the physiological roles of NO include modulation of vasodilation^31^, support of oral homeostasis^32^ and assisting exercise performance^33^ by increasing muscle force production^34^ and decreasing oxygen and adenosine triphosphate consumption ^35^.

In addition to NOS-mediated endogenous production, exogenous NO can be generated via the enterosalivary nitrate (NO_3_^-^)-nitrite (NO_2_^-^)-NO pathway^36^. Dietary NO_3_^-^, derived mainly from plant-based sources, enters the circulation^37^ and around 25% is actively secreted into the saliva^38^ where a proportion is reduced to NO_2_^-^ by commensal NO_3_^-^-reducing bacteria. These bacteria, from multiple genera, including *Rothia, Neisseria*, *Kingella*, *Actinomyces* and *Veillonella*^32^ reduce NO_3_^-^ to NO_2_^-^. Specific bacteria, including strains of tongue-associated *Rothia mucilaginosa,* have been found to demonstrate superior NO_3_^-^-reducing capacity compared to other species^39^. Swallowed NO_2_^-^ is either converted to NO in the acidic environment of the stomach^40^ or resorbed into the circulation^38^. Circulatory NO_2_^-^ can be stored in various tissues, including the blood, heart and liver^41–45^ or reduced to NO as required via multiple pathways involving haemoglobin, myoglobin and xanthine oxidoreductase under hypoxic conditions^37^.

There is some preliminary evidence to suggest that exercise training alters the oral microbiome. For example, *Rothia* was found to be increased in abundance among professional rugby players, alongside an increase in the relative abundance of the genus *Streptococcus* and the caries-associated species*Streptococcus mutans*^46^ and *Streptococcus sobrinus*^47^, as well as the lactic acid-generating *Streptococcus thermophilus*^48,21^ . *Veillonella*, another genus with NO_3_^-^-reducing capability that uses lactic acid as a carbon source, was found in increased abundance among competitive water polo players by Kalabiska et al.,^49^. Despite these differences in the microbiome and the increases in salivary NO_2_^-^ following periods of exercise training^50^, only one study has investigated links between oral NO_2_^-^ production capacity and aerobic fitness. Thomas et al.,^51^ found a positive association between V̇O_2peak_ and oral NO_2_^-^ generation capacity among trained participants, suggesting adaptations in microbiome behaviour in response to regular exercise training.

Previous studies comparing athletes and untrained controls analysed the salivary microbiome^21,49^, providing only an overview of bacterial cells shed from various oral sites^52^. In addition, the use of short-read 16 s rRNA sequencing limited accurate species-level analysis of microbiome differences^53^ This means that further research is needed to determine to what extent aerobic exercise training impacts the oral microbiome and environment, to determine if these changes alter NO bioavailability, complementing the adaptations already seen in NOS-mediated NO generation, or alter dental disease risk.

The aim of this study was to compare the composition of the microbiome of the tongue dorsum and supragingival plaque between highly trained athletes who took part in competitive sports and untrained controls who failed to meet WHO physical activity guidelines, using PacBio long-read 16S rRNA sequencing. In addition, the effect of long-term differences in activity level on NO_3_^-^ and NO_2_^-^ bioavailability in blood and saliva, on salivary pH and on oral NO_2_^-^ production capacity (a proxy for NO_3_^-^ reduction capacity) were also investigated.

## Methods

### Participants

Ten highly trained competitive athletes and 10 untrained individuals were recruited from the local community. Inclusion criteria for the trained group were as follows: completion of moderate-vigorous aerobic exercise training, which totalled ≥ 6 h per week for ≥ 6 months prior to enrolment, placing participants in Tier 3 of the Participant Classification Framework proposed by McKay et al.,^54^, and completion of exercise training specifically aimed at improving performance in a competitive sporting event for ≥ 6 months. Untrained participants carried out < 1 h of organised aerobic exercise per week in the 6 weeks leading up to study participation. All participants reported good systemic and oral health, did not smoke, did not take regular medication, and had not been given antibiotic therapy or used antimicrobial mouthwash within 3 months of enrolment.

### Experimental design

**Fig. 1:**
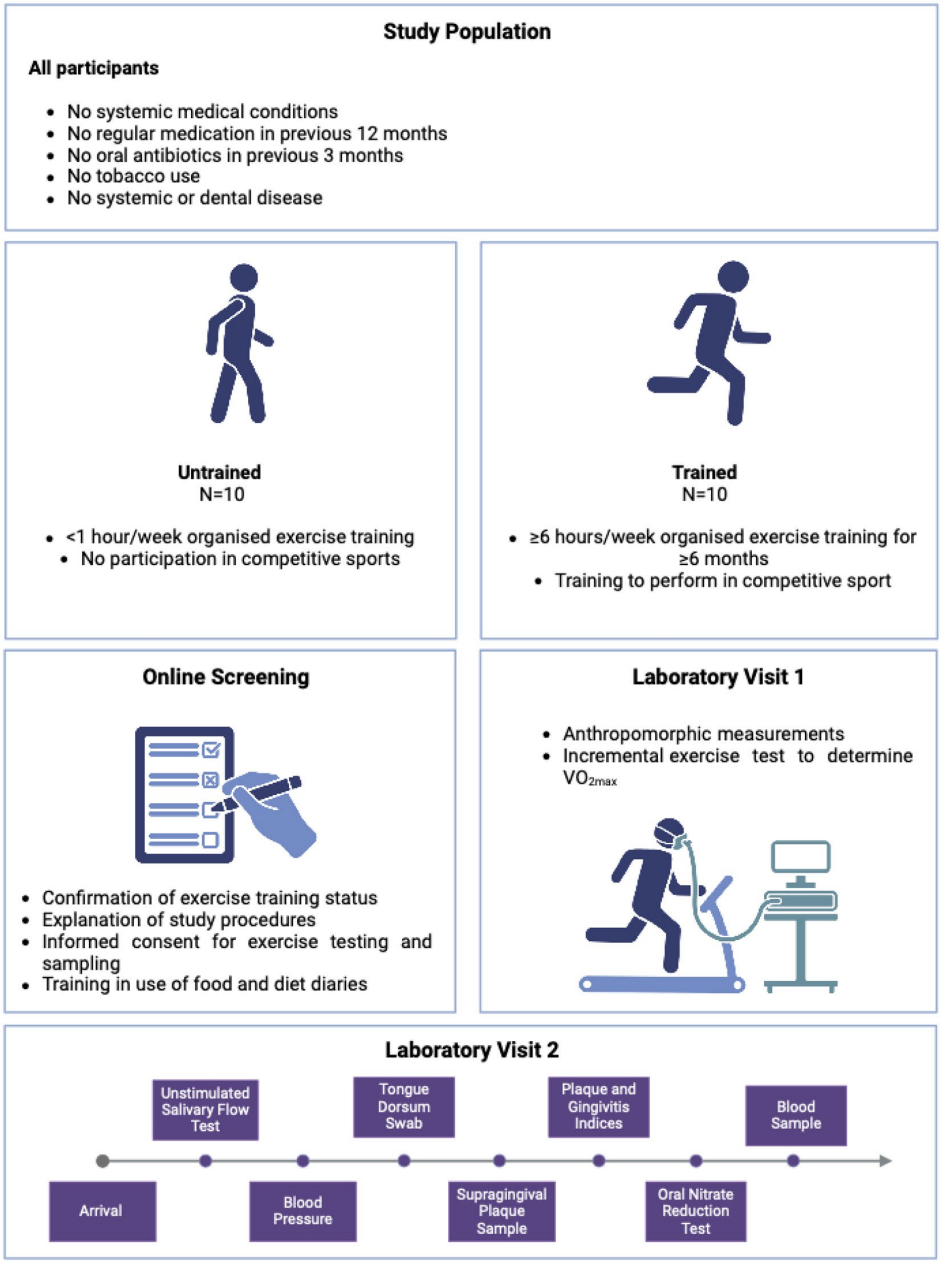
Shows a schematic of the study’s data collection procedures. Created in Biorender.com.

A schematic of the study visits is shown in Fig. 1. Participants attended the laboratory twice. In the first visit, anthropometric measurements were recorded, and an incremental exercise test to exhaustion was completed. Prior to the second lab visit, participants were instructed to refrain from carrying out tooth brushing or any other form of oral hygiene, as well as consuming caffeine or alcohol, for at least 12 h. They were asked to begin fasting 3 h before attending the lab. Compliance with fasting instructions was checked verbally. An hour prior to sampling, participants were advised to drink 500 ml of water. After this, participants did not consume any further liquids until sampling was complete. Samples of saliva, plasma, dental plaque, and a swab from the tongue dorsum were collected. Plaque and gingivitis indices were collected, and an oral NO_2_^-^ production capacity test was carried out. All participants filled out an exercise diary describing the duration and intensity of all exercise training sessions performed in the week prior to the first lab visit, as well as a food diary detailing the type and quantity of all food and beverages consumed in the week leading up to the second lab visit.

### Incremental exercise test

Participants warmed up at a self-selected pace for 10 min, then completed a graded exercise test to exhaustion on a motorised treadmill (Woodway, Waukesha, Wisconsin, USA). The incline was set to 1%. The belt speed was increased by 1 km/h each minute until 16 km/h was reached, the incline was then increased by 1% per minute until exhaustion. Heart rate was recorded by telemetry (Polar Electro, Oy, Finland). Expired gas was measured via indirect calorimetry (Jaeger/Vyntus, Vyaire Medical, IL, US) and analysed for respiratory variables to enable the calculation of V̇O_2max_, the maximum rate of oxygen uptake by an individual during exercise. The end criteria for the V̇O_2max_ were the achievement of at least one of the following: a plateau in oxygen uptake (< 150 ml/min or < 2.1 ml/kg/min) with an increase in work rate, a heart rate within 10 beats·min^-1^ of the age-adjusted theoretical maximum heart rate (220-age), a respiratory exchange ratio greater than 1.15 or an RPE score > 18.

### Dietary analysis

Total dietary NO_3_^-^ consumption was calculated as the sum of all NO_3_^-^ rich substances consumed during each meal, according to the weight recorded by the participants, multiplied by their estimated average NO_3_^-^ content^39^ Martin-Leon et al*.,* 2021).

### Saliva sampling

The resting salivary flow rate was measured using the passive drool technique^55^. In brief, participants sat upright for 5 min while refraining from swallowing. Saliva was allowed to drip into a collection container without external stimulation or active movement. The saliva was then weighed and centrifuged for 10 min at 13500 RPM prior to freezing at -80 °C.

### Blood pressure and mean arterial pressure

Systolic blood pressure (SBP) and diastolic blood pressure (DBP) were measured in triplicate using an automated cuff (Orman M6, Intelli-Sense. Hoofdorp, Netherlands) after the participant had remained supine for at least 30 min. The values were then averaged. If measurements > 120/80 mmHg were recorded, the patient was left for 5 min, and the blood pressure was re-recorded. Mean arterial pressure (MAP) was then calculated using the equation MAP = 2(DBP + SBP)/3.

### Plaque sampling

Supragingival plaque samples were collected from the mesiobuccal of teeth 16 and 26 using a sterile curette. Samples were immediately transferred to a sterile Eppendorf containing 500 μL of sterile phosphate-buffered saline. Plaque biofilm cells were harvested via non-refrigerated centrifuge (13,500 RPM for 10 min) and excess supernatant discarded prior to freezing. Samples from the tongue dorsum were collected using a sterile hydraflock (Puritan HydraFlock Swabs, Puritan Diagnostics LLC, Guilford, Maine, USA), rolled along the posterior surface for one minute. Tongue dorsum samples were stored in 150 μL of glycerol and 850 μL of sterile saline.

### Gingival bleeding indices

Signs of gingival bleeding were assessed visually and using a sterile CPITN C probe, moved around the gingival margin (Perfection Plus, Southampton, England). The upper and lower 8 s were excluded from analysis. Each tooth was split into six sites (mesiobuccal, mid-buccal, distobuccal, mesiolingual, mid-lingual and distolingual). The presence of bleeding from the gingival margin was recorded at each site, and the percentage of total sites with bleeding was calculated.

### Blood sampling

A venepuncture needle was inserted into the antecubital vein of the arm. Venous blood was collected into vacutainer tubes containing EDTA (BD vacutainer K2E 7.2 mg, Plymouth, U.K.) and centrifuged at 4000 rpm for 10 min. The plasma was transferred to an Eppendorf prior to freezing.

### Oral NO2- production test

Participants rinsed their mouth with a solution of 80 μmol sodium NO_3_^-^ dissolved in 10 mL of ultrapure water, reflecting the tenfold higher salivary NO_3_^-^ observed following a NO_3_^-^-rich meal. The solution was held in the participant’s mouth for five minutes before being expelled into a clean container. The reduced solution was spun at 13,500 RPM for 5 min and stored prior to absolute NO_2_^-^ determination.

### NO2- analysis

A solution of 2.5 ml acetic acid, 0.5 ml of deionised water and 25 mg sodium iodide was placed into a glass purge vessel heated to 50 °C and connected to a NO analyser (Sievers NOA 280i, Analytix, UK). A standardisation curve was constructed by injecting 100 μL of NO_2_^-^ solution to achieve a maximum concentration of 3000 μM. Samples were thawed at 37** °C** in a water bath and 100 μL of thawed sample was injected into the purge vessel. Saliva and oral NO_2_^-^ production test samples were diluted with deionised water at a ratio of 1:100 prior to injection. The NO_2_^-^ content of each sample was calculated using the area under the standard curve (AUC). In addition to absolute quantification, the salivary NO_2_^-^ levels were normalised by dividing the absolute concentration by the salivary flow rate.

### NO3- analysis

Vanadium reagent (24 mg of vanadium tri-chloride and 3 ml of 1 M hydrochloric acid) was added to the purge vessel and heated to 90 °C. A standard curve was created by injecting 50 μL NO_3_ solution at concentrations up to 100 μM into the purge vessel. Plasma samples were de-proteinised by adding and vortexing 100 μl of sample alongside 200 μl of solutions of ZnSO_4_ and NaOH (both in deionised water at a 1:1 ratio), prior to centrifuging for 5 min at 4000 RMP. Saliva and samples were diluted with deionised water at a ratio of 1:100 prior to injection. 50 μL of sample was injected into the purge vessel and the NO_3_^-^ content was calculated as described above. In addition to absolute quantification, the salivary NO_3_^-^ levels were normalised by dividing the absolute concentration by the salivary flow rate.

### pH analysis

Salivary pH was measured in duplicate using a circular electrode pH-meter (1140 Mettler Toledo, Greisensee, Switzerland). The pH value was recorded after an unchanged pH value was observed for a period of at least 7 s. The pH meter was calibrated before analysis and after every 10 samples using buffers with known pH (4.01 and 7.00). The electrode was rinsed with 18 Ω deionised water between samples.

### DNA isolation

DNA isolation was carried out according to manufacturer’s instructions using the MasterPure Complete DNA and RNA Purification Kit (Epicentre Biotechnologies, Madison, WI, USA). An additional treatment step with 20 mg/mL lysozyme was included for all samples (Sigma-Aldrich, St-Louis, MO, USA). The DNA concentration of each sample was checked using the Qubit 1 × dsDNA HS Assay Kit and a Qubit 3 Fluorometer (both Thermo Scientific, Waltham, Massachusetts, USA), according to manufacturer’s instructions. Samples were frozen prior to sequencing.

### Bacterial 16S rRNA sequencing

SMRT Amplicon sequencing of the 16S rRNA gene was carried out with a PacBio Sequel II sequencer according to the manufacturer’s instructions (PacBio, California, USA). The functions in the DADA2 R package (^56,57^, R version 4.1.0 and DADA2 version 1.20, were used to process the raw PacBio Circular Consensus Sequencing (CCS) reads in each sample to generate an abundance table of chimera-free amplicon sequence variants (ASVs). First, primers were removed from the raw reads. Next, reads shorter than 1000 or longer than 1600 bases, with more than 5 expected errors or with any ambiguous N base were removed. Subsequently, the error rates for each base transition were estimated, with “PacBioErrfun” error estimation function and 32 banding size. Dereplication was carried out to combine all identical reads into unique sequences, with abundance equal to the number of reads combined. Taking the dereplicated reads and the error estimations, sequence variants were inferred. The variants reconstructed by combining a left-segment and a right-segment from two more abundant sequences, with “MinFoldParentOverAbundance” parameter equal to 3.5, were identified as chimeric and discarded, to obtain the final ASVs. Finally, taxonomy was assigned to each ASV by means of the naive Bayesian classifier method, with bootstrap confidence equal or greater than 80, using as reference the Silva species training set, version 138.1 ^58–60^.

### Statistical analysis

The physiological and exercise data were analysed in GraphPad PRISM and the microbiome data were analysed in R as described below. Physiological and exercise data were tested for normality using the Shapiro–Wilk test and by visual examination of box plots. Non-parametric tests were used where appropriate. Differences between groups were analysed using independent samples t-tests, Wilcoxon tests or Fisher’s exact tests. Parametric data are displayed as mean ± SD and non-parametric data as median (IQR).

For microbial composition analysis, R programming language was used^57^,R Core Team, 2021). Rarefaction curves were calculated using Vegan library, version 2.6–4^61^. Alpha diversity matrices were calculated using the minimum number of reads annotated at the species level in a sample (4900 reads/sample). Adonis tests (Permutational Multivariate Analysis of Variance Using Distance Matrices or PERMANOVA) and constrained correspondence analysis (CCA) were used to assess differences in beta diversity [70] Prior to conducting the Adonis tests, the assumption of homogeneity of group dispersions was tested and confirmed using “betadisper”, indicating that observed differences in beta diversity observed between the trained and untrained groups were not attributable to differences in dispersion among groups. Statistical differences in individual sequences at the species and genus level between groups were calculated using Wilcoxon tests on the relative abundance values following normalisation of the data with Analysis of Compositions of Microbiomes with Bias Correction (ANCOM-BC2)^62^. *P*-values were corrected for multiple testing using the false discovery rate (FDR) method. Taxons were filtered by abundance (taxa included in the analysis if the average abundance > 0.15%) and prevalence included if the abundance value closest to zero, multiplied by four, was present in 70% of samples) when compared between groups.

Association heatmaps were constructed by computing pairwise associations between bacterial taxa and other parameters based on a multivariate approach described by González et al.,^63^, using the ‘mixOmics’ R package^64^, ^65,66^), version 6.22.0. In brief, associations were obtained between bacterial sequences and other parameters by the projection of variables onto a correlation circle plot^65^ derived from a principal component analysis. Only negative associations below -0.4 and positive associations above 0.4 are shown on the heatmaps. To complement these associations, Spearman’s rho correlations between parameters were determined, alongside adjusted* p*-values using the cor.test function of the stats library of R. To further minimise the risk of false positive results, only associations confirmed by Spearman’s rho correlations were discussed.

## Results

### Baseline characteristics

Table 1 shows the demographic characteristics of the subjects. The control group were significantly heavier compared to the trained athlete group (body mass, *p* < 0.02 and Body Mass Index (BMI), *p* < 0.002) and had significantly higher DBP (p < 0.0003). No other significant differences were detected. Table 2 shows the dental characteristics of the participants. No significant differences were found between groups, except in salivary flow rate, which was lower in the trained group (*p* < 0.04).

**Table 1:** Demographic characteristics of the participants. Data shown as mean ± SD or median (IQR). ^∅^Unpaired t-test, ^⊕^Fisher’s exact test ^⊗^Wilcoxon test.

Parameter	Trained N = 10	Untrained N = 10	*p*-value
Demographic characteristics			
Female (n)	3	4	
Age (y/o)	30 ± 10	31 ± 7	0.75^∅^
Body mass (kg)	63.4 ± 9.6	81.8 ± 20.4	0.02^∅^
Height (cm)	172 ± 9	172 ± 9	0.10^∅^
BMI (kg/m^2^)	21.3 (19.7—22.9)	25.0 (23.7—31.1)	0.003^∅^
SBP (mm Hg)	116 ± 8	122 ± 13	0.4^⊗^
DBP (mm Hg)	69 ± 7	83 ± 11	0.0003^⊗^

**Table 2:** Dental characteristics of the participants. Data shown as mean ± SD. ^∅^Unpaired t-test, ^⊕^Fisher’s exact test ^⊗^Wilcoxon test.

Parameter	Trained N = 10	Untrained N = 10	*p*-value
Seen dentist within 12 months (%)	60	40	0.66^⊕^
Interdental cleaning aids used (%)	40	70	> 0.99^⊕^
Gingival index (%)	4.3 ± 4.2	3.5 ± 5.1	0.60^⊗^
Salivary flow rate (ml/min)	0.3 ± 0.2	0.6 ± 0.4	0.04^∅^
Estimated weekly NO_3_^-^ consumption (mg/kg)	177 ± 92	188 ± 99	0.81^∅^

### Participant training status

**Fig. 2:**
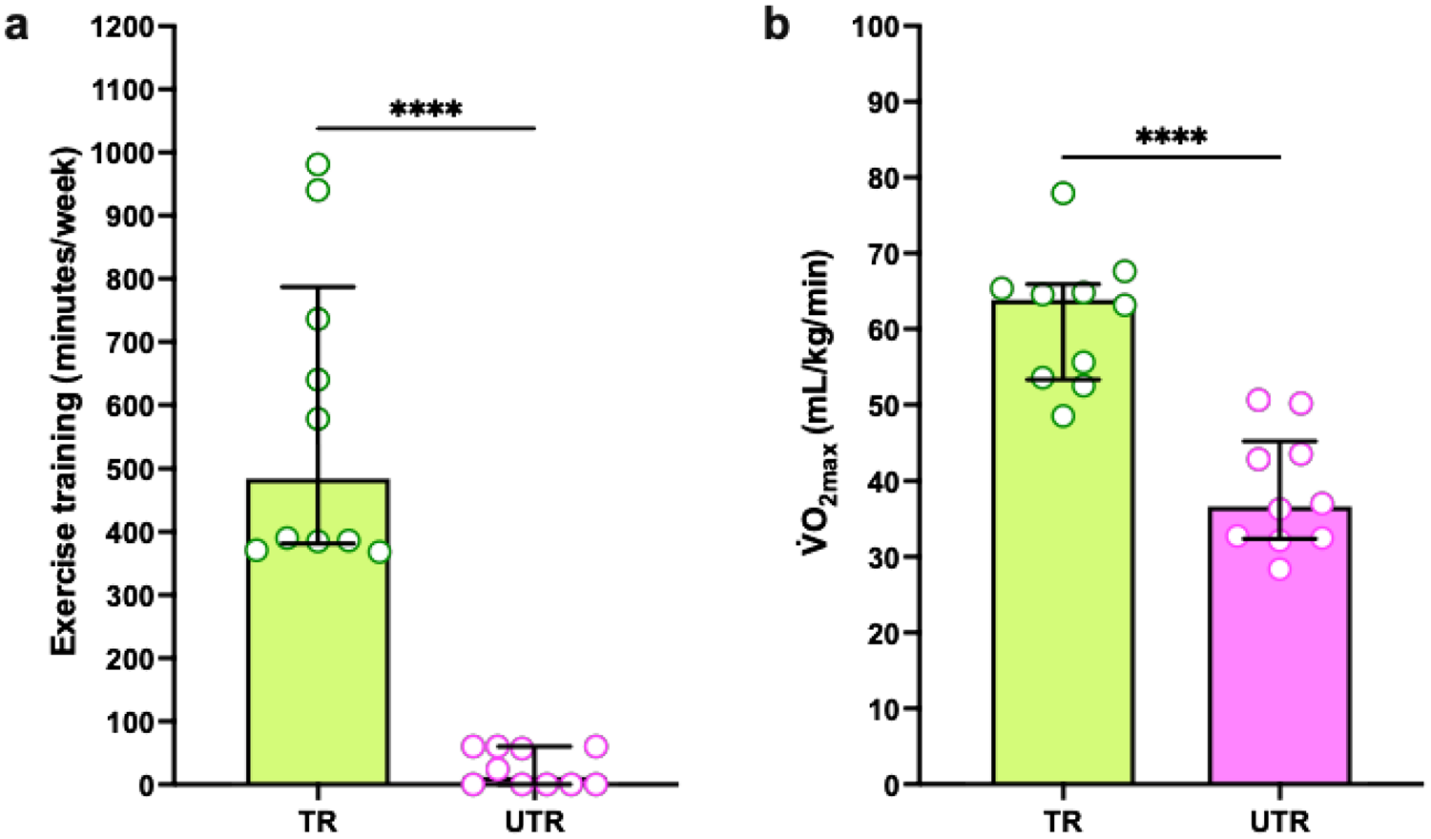
Comparison of training status and aerobic fitness between the trained (TR) and untrained (UTR) participants. (**a**) Time spent carrying out exercise training in the week before testing^⊗^. (**b**) Relative V̇O_2max_^∅^ . Each point represents a participant in the study. N = 10 for each group. ^⊗^Wilcoxon test with data shown as median with IQR, ^∅^Unpaired t-test with data shown as mean ± SD. **** *p* < 0.0001.

The trained group engaged in more cumulative weekly exercise training compared to the untrained group (median 484, IQR 382–787 min per week vs. median 12, IQR 0–60 min per week), with a statistically significant between-group difference (Wilcoxon rank-sum test, W = 0, *p* < 0.0001), Fig. 2a. The trained group also demonstrated a significantly higher V̇O_2max_ (61.4 ± 8.8 vs. 38.6 ± 7.8 mL/kg/min), with a between-group difference of 22.7 mL/kg/min (t = 6.127, p < 0.001, 95% CI: 15.0–30.5). Figure 2b.

### Physiological levels of NO_3_^-^ and NO_2_^-^, salivary pH and Oral NO_2_^-^-production

**Fig. 3:**
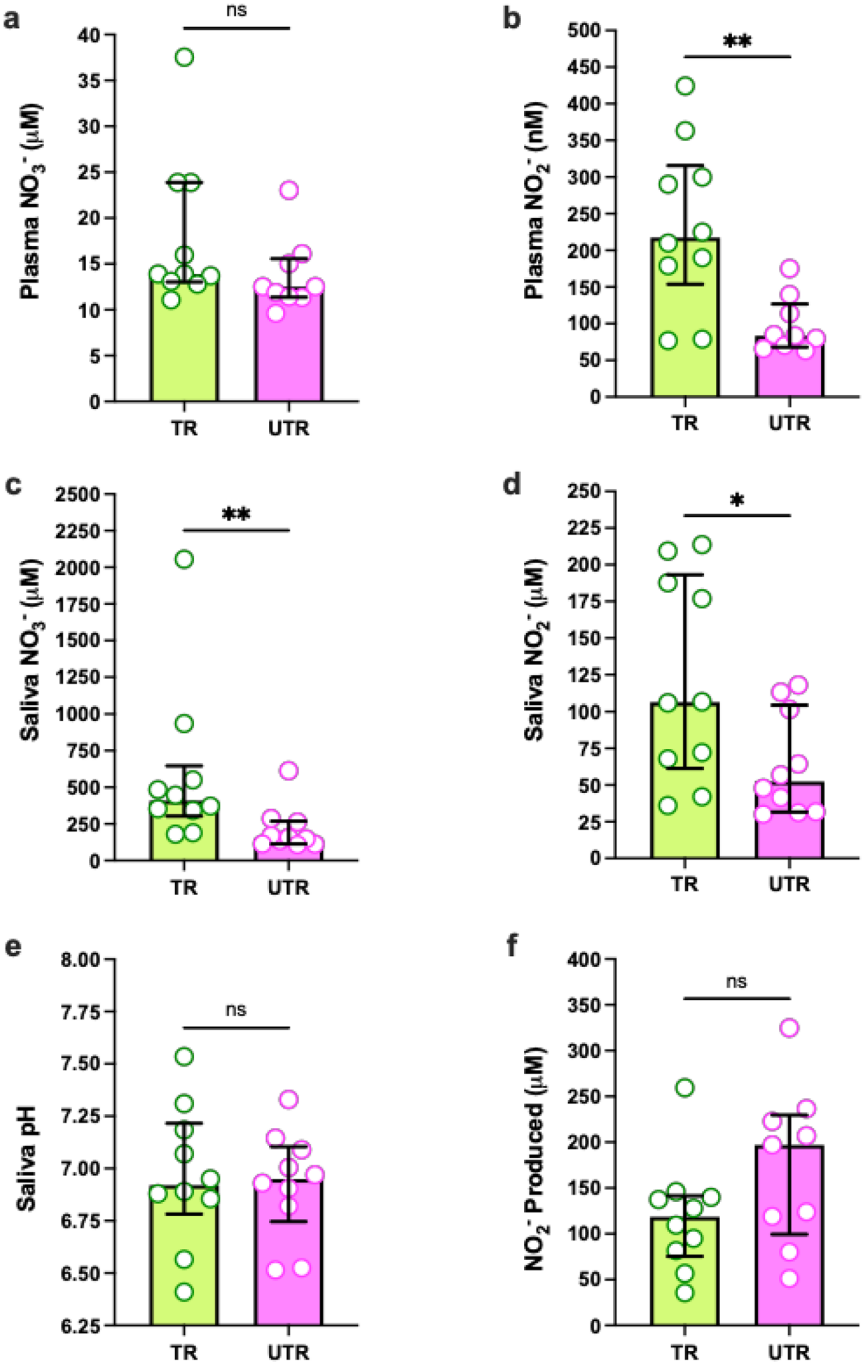
Differences in the saliva and plasma between groups and differences in weekly NO_3_^-^ consumption between trained (TR) and untrained (UTR) participants. (**a**) Plasma NO_3_^-⊗^. (**b**) Plasma NO_2_^-∅.^ (**c**) Salivary NO_3_^-⊗^. (**d**) Salivary NO_2_^-⊗^. (**e**) Resting salivary pH^∅^. (**f**) NO_2_^-^ produced following administration of a NO_3_^-^ mouth rinse^∅^. Each point represents a participant in the study. N = 10 for each group for salivary values, N = 9 for the untrained group for plasma values. and N = 9 for both groups for the dietary NO_3_^-^ consumption estimate. ^⊗^Wilcoxon test with data shown as median with IQR, ^∅^Unpaired t-test with data shown as mean ± SD. * *p* < 0.05, ** *p* < 0.01.

No significant difference in plasma NO_3_^-^ concentration was found between groups (Wilcoxon rank-sum test, W = 26, p < 0.13, Fig. 3a). Plasma NO_2_^-^ concentration was significantly higher in the trained group (t = 3.439, *p* = 0.003, 95% CI 52.7—220.0 nM, Fig. 3b).

Significantly higher levels of both salivary NO_3_^-^ (Wilcoxon rank-sum test, W = 12, *p* = 0.02, Fig. 3c) and NO_2_^-^ (t = 2.377, *p* = 0.03, 95% CI 6.27—109.6 uM, Fig. 3d) were observed in the trained group. Following correction for the salivary flow rate, these results remained unchanged (Wilcoxon rank-sum test, W = 9, *p* = 0.002 for NO_3_^-^ and unpaired t-test, t = 3.21, *p* = 0.005). In contrast, no significant differences were detected in the resting salivary pH (t = 0.3128, *p* = 0.76, 95% CI -0.3—0.2, Fig. 3e) or NO_2_^-^ produced following the administration of a NO_3_^-^-rich mouth rinse (t = 1.597, *p* = 0.13, 95% CI -17.5—126.8uM, Fig. 3f).

### Microbiome results

**Fig. 4:**
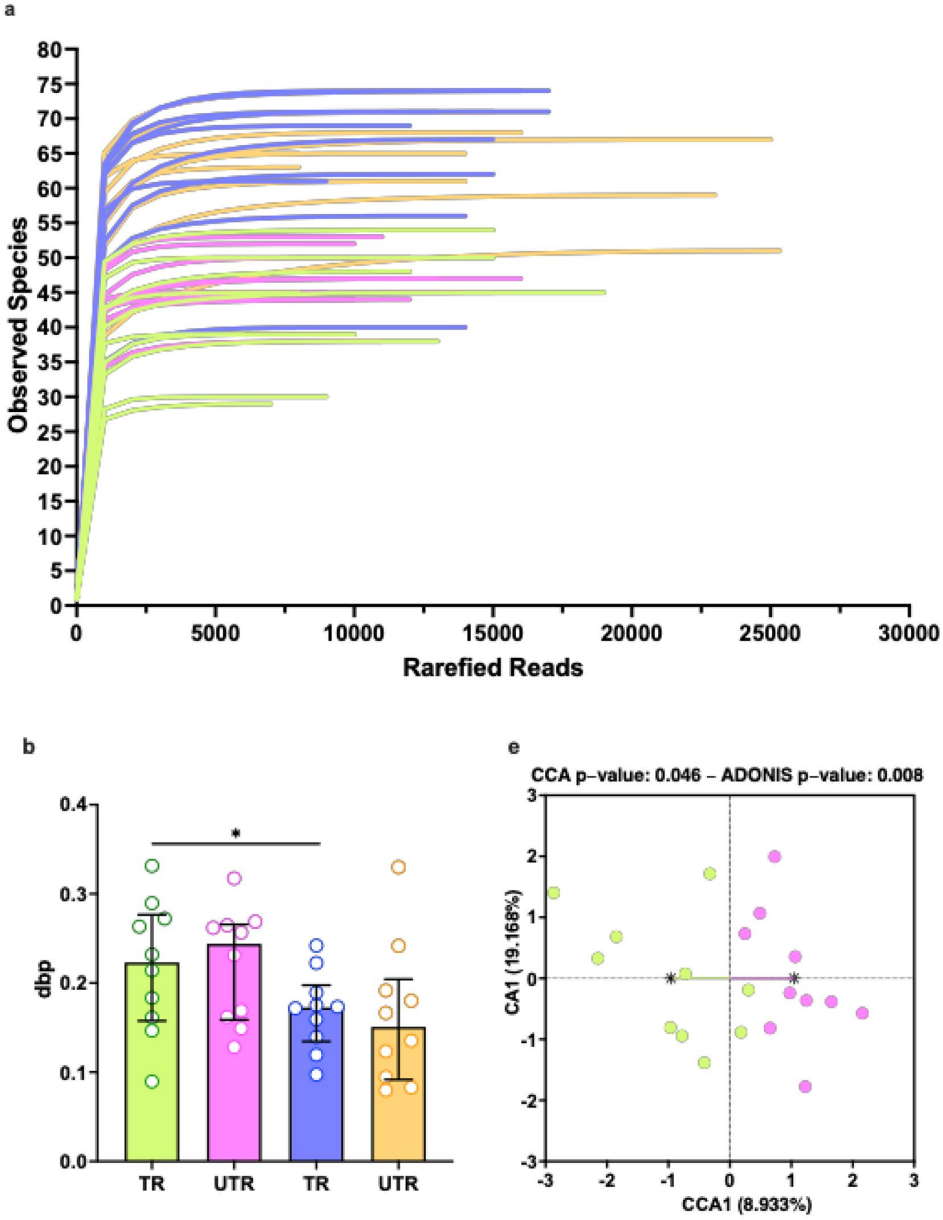

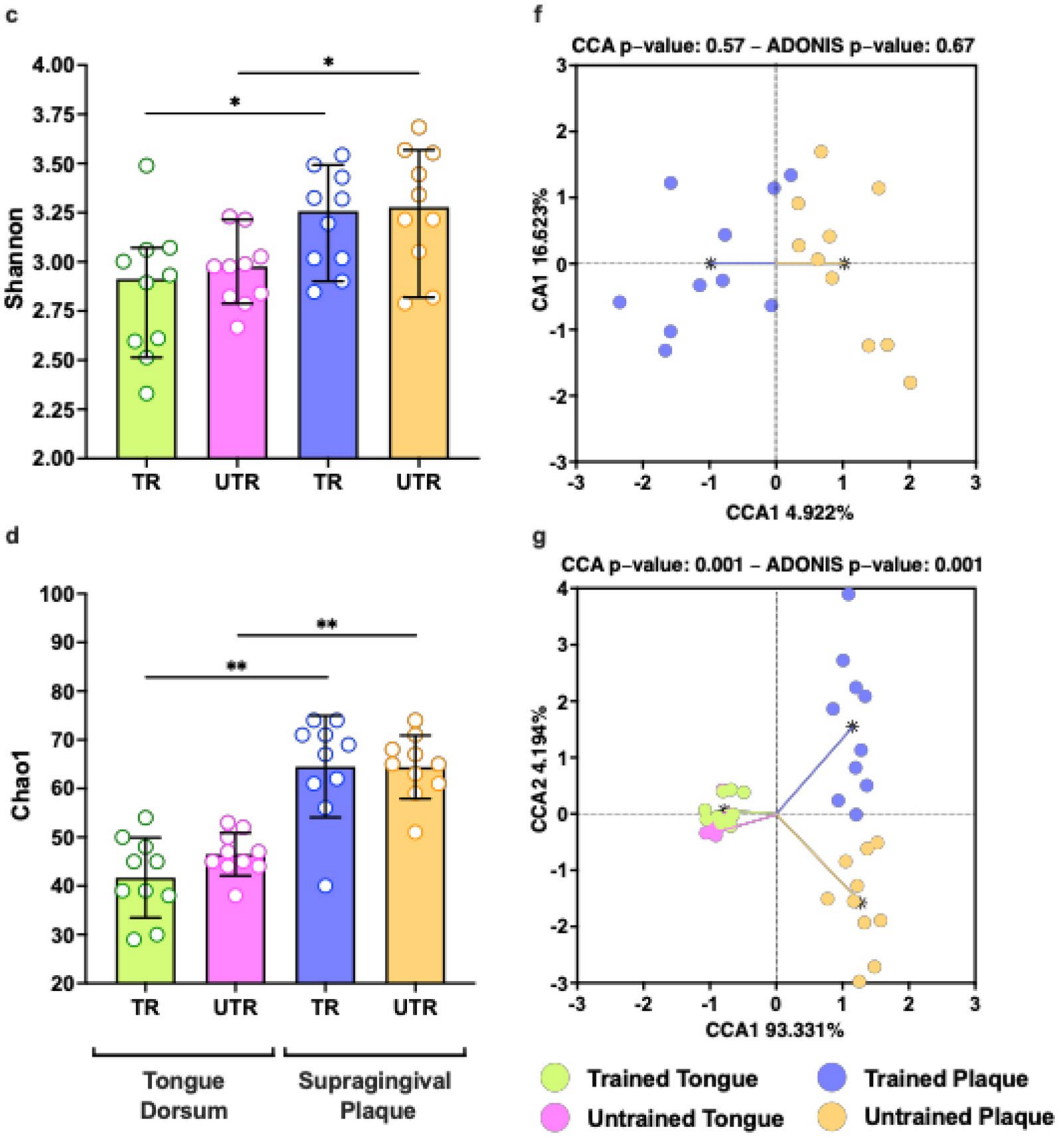

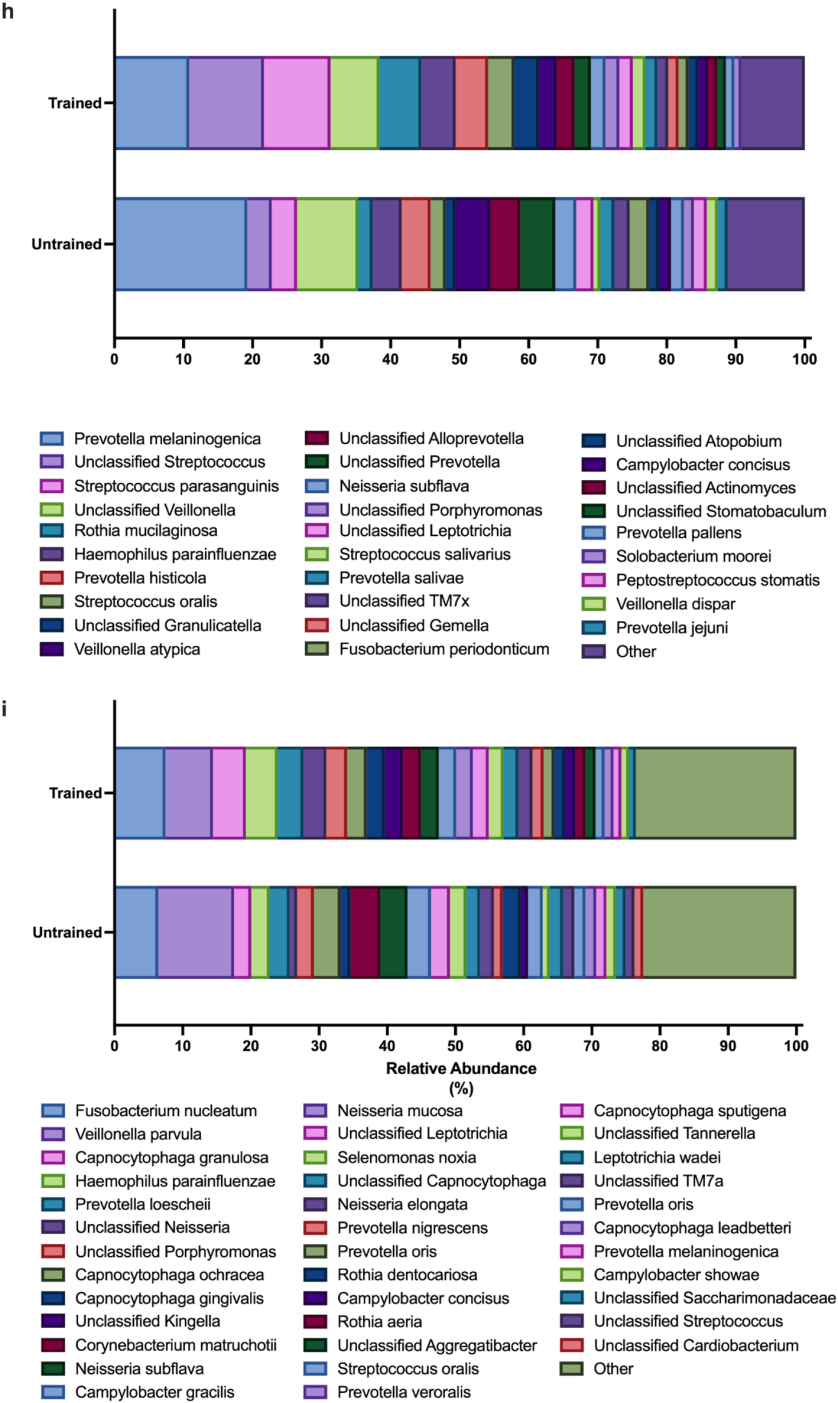
Assessing the impact of long-term intensive aerobic exercise training on the diversity and composition of the oral microbiome. (**a**) Rarefaction curves for observed species. Differences in alpha diversity indices (**b**) dbp. (**c**) Shannon index. (**d**) Chao1 index between trained (TR) and untrained (UTR) participants^⊗^ and between the microbiome of the tongue and subgingival plaque in participants of the same training status^∅^. Data shown as median with IQR. Differences in beta-diversity between the microbiome of trained and untrained participants (**e**) Tongue dorsum. (**f**) Subgingival plaque. (**g**) Combined differences in beta-diversity between microbiome samples of different groups. Most abundant species in the microbiome of trained and untrained participants (**h**) Tongue dorsum. (**i**) Supragingival plaque in the trained and untrained groups. All species with relative abundance > 1% are shown, with the remaining species described as ‘other’. ^⊗^Wilcoxon test .^∅^Wilcoxon signed-rank test. * *p* < 0.05, ** *p* < 0.01.

Sequencing of the 20 samples collected from the tongue dorsum samples yielded a median of 11,182 reads per sample (ranging from 5544 to 18,648 reads) following the DADA2 pipeline, including quality filtering and trimming. After removing taxa with a low abundance and prevalence, these reads were classified into 30 taxa on a genus-level and 52 taxa on a species-level. The samples of supragingival dental plaque collected from the mesiobuccal sites of teeth 16 and 26 were combined for analysis. Sequencing yielded a median of 14,984 reads per sample (ranging from 8319 to 25,783 reads) following the DADA2 pipeline. These reads were classified into 42 taxa on a genus-level and 87 taxa on a species-level after removing low-abundance and prevalence taxa. Rarefaction curves suggested taxonomic diversity was fully covered, with 4900 reads per sample Fig. 4a

To evaluate differences in alpha diversity, the distinct phylogenetic branches (dpb) (Fig. 4b), Shannon (Fig. 4c), and Chao1 (Fig. 4d) indices were calculated. No significant differences were found between groups in the microbiome of the tongue dorsum (all p > 0.09) or supragingival dental plaque (all *p* > 0.53). However, significant differences were detected in all 3 indices between samples collected from the different oral sites (all *p* < 0.04).

To investigate whether regular exercise training altered the microbial community structure, beta diversity analysis was conducted. A significant difference was found between the bacterial community of the tongue dorsum between the trained and untrained groups as assessed using CCA and an Adonis (PERMANOVA) test (both *p* < 0.046, Fig. 4e). No difference was detected in the beta diversity of the supragingival plaque samples (both *p* > 0.57, Fig. 4f). While the microbiome beta-diversity of samples collected from the tongue dorsum and supragingival plaque was also found to be significantly different (both *p* < 0.001, Fig. 4g), the groups also showed significantly different dispersions (*p* < 0.001), which reduces the reliability of the PERMANOVA result.

The high-fidelity long-read 16 s rRNA sequencing used enabled accurate quantification of the composition of the oral microbiome at the species level. The most abundant species within the niches of the tongue dorsum and supragingival plaque are shown in Fig. 4i and Fig. 4j. *Prevotella melanginogenica* was the most abundant species on the tongue dorsum in both groups. The next most abundant species in the trained group were unclassified *Streptococcus, Streptococcus parasanguinis*, unclassified *Veillonella* species and *R. mucilaginosa.* In the untrained group, the next most abundant species found on the tongue dorsum were unclassified *Veillonella, Veillonella atypica,* unclassified *Prevotella* species, unclassified *Alloprevotella* species and *Haemophilus parainfluenzae.* In the supragingival plaque, the two most abundant species were *Fusobacterium nucleatum* and *Veillonella parvula,* followed by *Capnocytophaga granulosa*, *Haemophilus parainfluenzae* and *Prevotella loescheii* in the trained group and *Corynebacterium matruchotii*, *Neisseria subflava* and *Capnocytophaga ochracea* in the untrained group.

At the genus level, similar differences in alpha diversity and beta diversity were observed. Differences were seen between sites for the dpb (all *p* > 0.013, Supplementary Fig. 1b), Shannon (all *p* < 0.014, Supplementary Fig. 1c), and Chao1 (all *p* < 0.03, Supplementary Fig. 1d) indices (all *p* < 0.014). A difference in beta diversity of the tongue dorsum microbiome was detected between the trained and untrained groups (CCA *p* = 0.033, Adonis *p* = 0.014, Supplementary Fig. 1e), while no difference was detected in the supragingival plaque (CCA *p* = 0.86, Adonis *p* = 0.88, Supplementary Fig. 1f.). At the genus level, the beta-diversity of the tongue dorsum and supragingival plaque was also found to be significantly different (both *p* < 0.001, Supplementary Fig. 1 g), however, as at the species level, the groups also showed significantly different dispersions (*p* < 0.04), which reduces the reliability of the PERMANOVA result.

*Streptococcus*, followed by *Prevotella* and *Veillonella* were the most abundant genera in both groups on the dorsal surface of the tongue (Supplementary Fig. 1 h). *Veillonella*, followed by *Capnocytophaga* and *Fusobacterium* predominated in the supragingival plaque (Supplementary Fig. 1i)*.*

### Individual genus and species-level differences

Wilcoxon tests of ANCOM-BC2 transformed relative abundance data were used to detect differences in individual sequence abundance. To ensure the investigated genera and species were those which contributed most to the compositional differences seen in the oral microbiome in active individuals, a further abundance filter (the inclusion of only genera and species where the lowest abundance was at least ten times the lowest abundance above zero in > 60% of samples) was applied.

Following correction for multiple testing, no significant differences were detected at the genus level in the microbiome of the tongue dorsum (all *p* > 0.07) or in the supragingival plaque (all p > 0.98). Full results of the microbiome analysis can be found in Supplementary File 2.

**Fig. 5:**
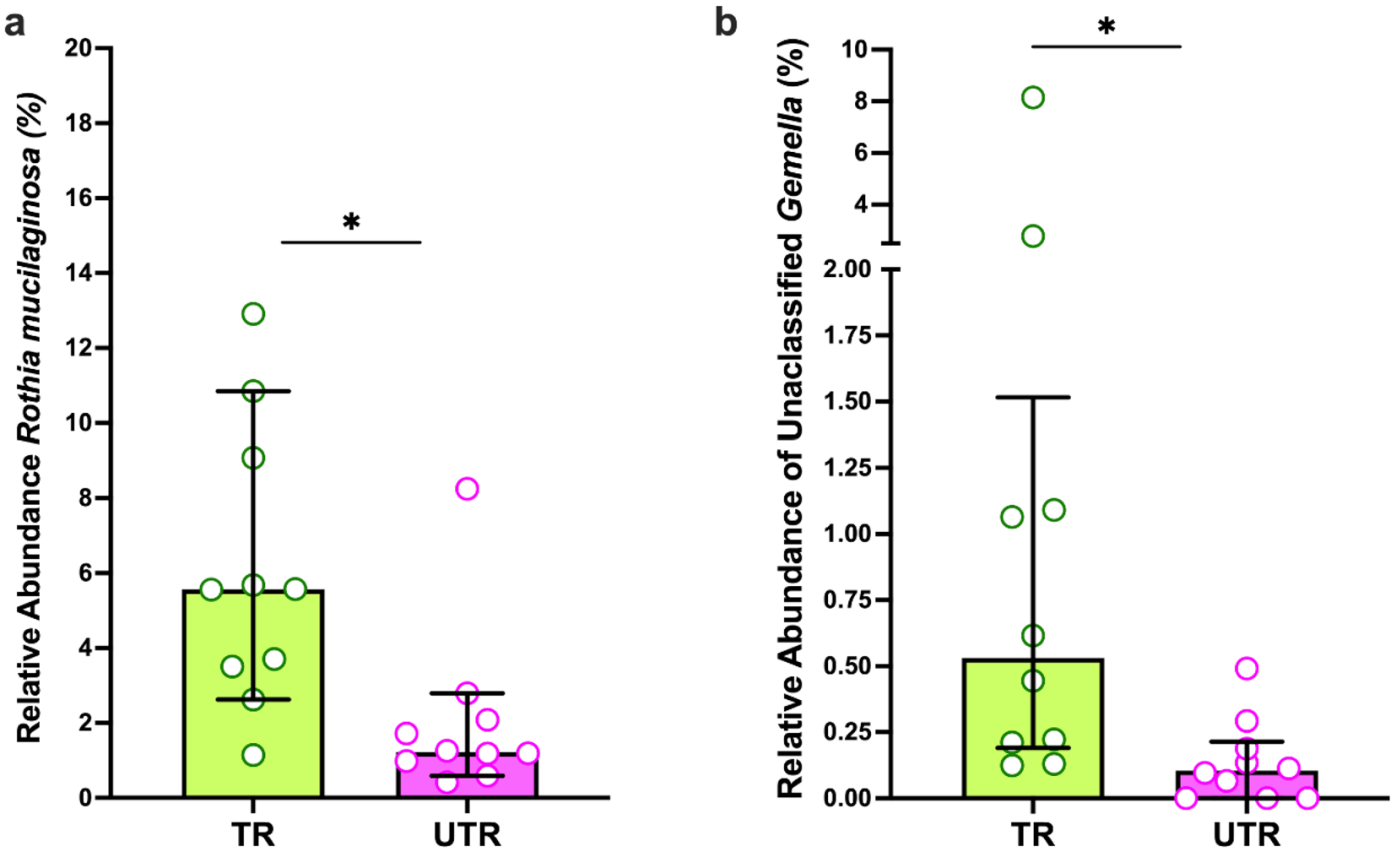
Shows significant species-level differences between the trained (TR) and untrained (URT) groups. (**a**) Relative abundance of *Rothia mucilaginosa.* (**b**) Relative abundance of unclassified *Gemella.* N = 10 for each group. Data shown as median with IQR. Each point represents a participant in the study. Groups were compared using a Wilcoxon test, carried out on the ANCOMBC-2 transformed relative abundance values. * *p* < 0.05.

Species-level differences were detected in the microbiome of the tongue dorsum between the trained and untrained groups. Following correction for multiple testing, the relative abundance of NO_3_^-^-reducing *R. mucilaginosa* and unclassified species assigned to the genus *Gemella* were significantly higher in the trained group (both *p* < 0.04, Fig. 5a,b)*.* In the supragingival plaque, no significant differences at the species levels were detected between groups following correction for multiple testing (all *p* > 0.91). Full results of the microbiome analysis can be found in Supplementary File 2.

Bacteria were grouped as NO_2_^-^-producing species or NO_3_^-^-reducing species according to^32^, as described in Supplementary Tables 1 and 2. No significant between-group difference was observed in the summed relative abundance of all known NO_2_^-^-producing species or NO_3_^-^-reducing species of the tongue dorsum or supragingival plaque (both *p* > 0.80, Supplementary Fig. 2).

### Associations between the oral microbiome and regular exercise training^-^

An apparent relationship was detected between the microbiome of the tongue dorsum and regular exercise training. To further investigate this, associations between the abundance of oral bacteria at the genus and species level, physiological concentrations of NO_3_^-^ and NO_2_^-^, V̇O_2max_ and the volume of training carried out were assessed by calculating Spearman’s correlation coefficients.

**Fig. 6:**
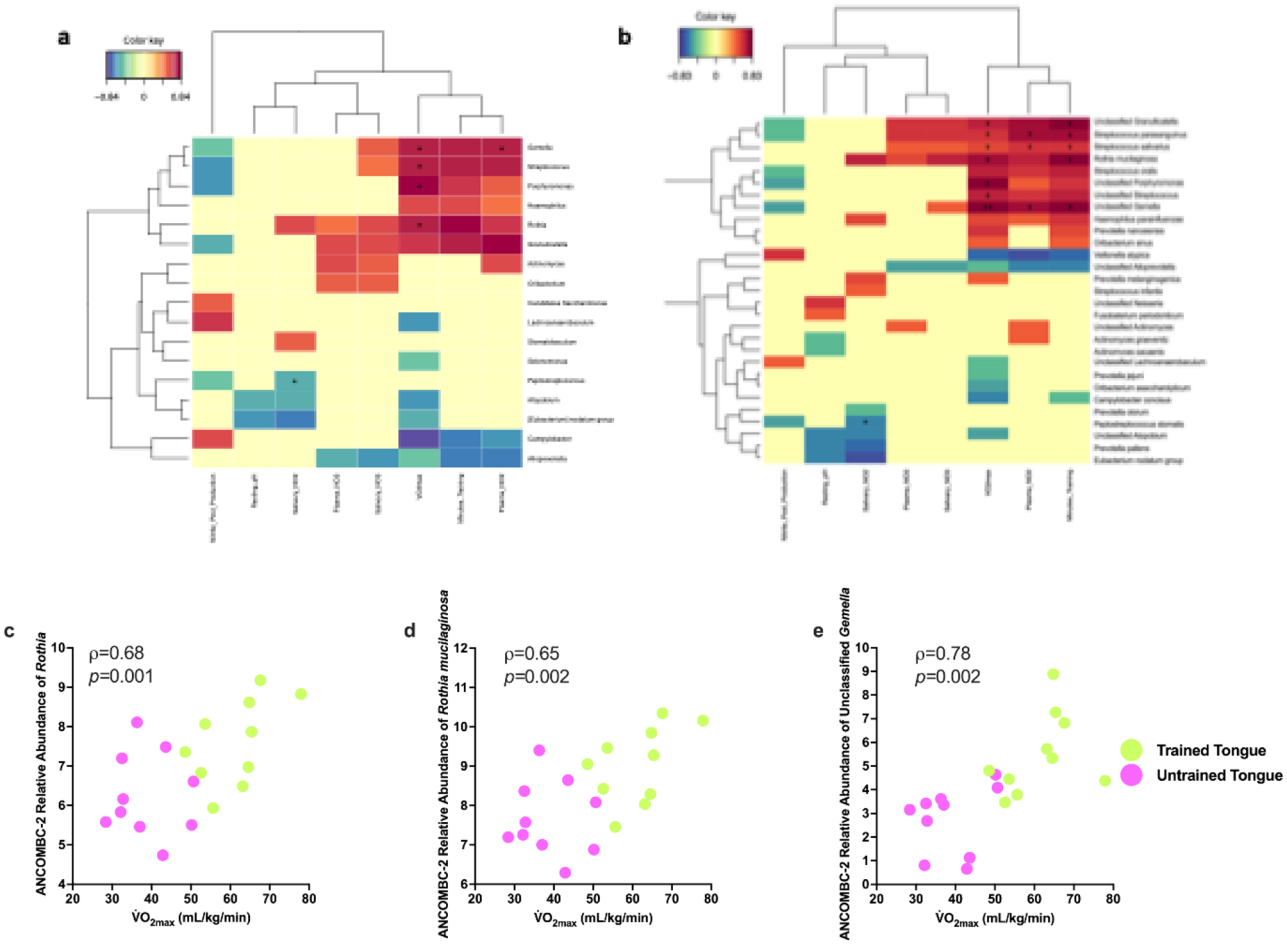
Associations between the composition of the tongue dorsum microbiome, NO_3_^-^ and NO_2_^-^ levels and the aerobic training status of all participants. Heatmaps with associations between markers of exercise training status, NO_3_^-^ and NO_2_^-^ levels, salivary pH and oral NO_2_^-^ production levels and bacterial abundance at (**a**) the genus level and (**b**) species level. These heatmaps show the association between bacterial abundances after ANCOM-BC2 transformation and other parameters, which were obtained based on their projection onto a correlation circle plot derived from a principal component analysis. The correlation circle plots are shown in Supplementary Fig. 4. Only negative associations below -0.4 and positive associations above 0.4 between species are shown. To complement the association heatmaps, correlations between species and other parameters were determined with Spearman’s rho and marked with asterisks on the heatmap (* *p*-adjusted < 0.05, ** *p*-adjusted < 0.01). Scatterplots showing the V̇O_2max_ and ANCOM-BC2 transformed relative abundance of (**c**) *Rothia*. (**d**) *R. mucilaginosa.* (**e**) Unclassified *Gemella* species.

Multiple significant correlations between V̇O_2max_ and training volume and the tongue dorsum microbiome were detected. Genus level associations are shown in Fig. 6a. At the genus level, *Gemella* (ρ = 0.73*, p* = 0.02)*, Porphyromonas* (ρ = 0.68*, p* = 0.03), *Rothia* (ρ = 0.65*, p* = 0.03) and *Streptococcus* (ρ = 0.62*, p* = 0.03) all positively correlated with the V̇O_2max_. No significant correlations were found with the supragingival plaque microbiome (all *p* > 0.09).

Species-level associations are shown in Fig. 6b. Positive correlations were detected between the V̇O_2max_ and the relative abundance of unclassified *Gemella* species (ρ = 0.80*, p* = 0.002), *R. mucilaginosa* (ρ = 0.68*, p* = 0.02), unclassified *Porphyromonas* species (ρ = 0.62*, p* = 0.03), *Streptococcus salivarius* (ρ = 0.60*, p* = 0.03), *S. parasanguinis* (ρ = 0.60*, p* = 0.04), an unclassified *Granulicatella* species (ρ = 0.59*, p* = 0.04) and unclassified *Streptococcus* species (ρ = 0.57, *p* = 0.04). An increased volume of exercise training also positively correlated with the relative abundance of unclassified *Gemella* species (ρ = 0.66*, p* = 0.02), *R. mucilaginosa* (ρ = 0.63*, p* = 0.03), unclassified *Granulicatella* species (ρ = 0.60*, p* = 0.04), *S. salivarius* (ρ = 0.57*, p* = 0.04) and *S. parasanguinis* (ρ = 0.55*, p* = 0.04). Positive correlations were detected between bacterial species found on the tongue dorsum and plasma NO_2_^-^. These were between unclassified *Gemella* species (ρ = 0.69, *p* = 0.001), *S. salivarius* (ρ = 0.67, p = 0.03) and *S. parasanguinis* (ρ = 0.67, p = 0.05). Significant correlations were also detected between salivary NO_2_^-^ and the genus *Peptostreptococcus* (ρ = *-*0.57*, p* = 0.05) and salivary NO_2_^-^ and the species *Peptostreptococcus stomatitis* (ρ = *-*0.62*, p* = 0.03). No significant correlations were found with the supragingival plaque microbiome at the species level (all *p* > 0.06).

The genus *Rothia* has been associated with participation in exercise training in previous studies^21,67^, while unclassified *Gemella* species and *R. mucilaginosa* were both found in higher abundance in the tongue microbiome of the trained group. The strong positive association between V̇O_2max_ and the relative abundance of each of these bacterial sequences is shown in Fig. 6c,e.

No significant correlations were found between the supragingival plaque microbiome and the volume of exercise training completed or the V̇O_2max_ (all *p* > 0.09 at the genus level and *p* > 0.06 at the species level). Complete results of the correlation analysis can be found in Supplementary File 2.

## Discussion

This is the first study to use long-read 16S rRNA sequencing to compare the species-level composition of the oral microbiome between highly trained competitive athletes and untrained controls. We did not detect differences between groups in the supragingival dental plaque, which suggests no link between consistent exercise training and the dental disease-associated microbiome at this site. Conversely, our data did suggest that the composition of the tongue microbiome of highly trained competitive athletes was significantly different to that of untrained individuals. This was accompanied by a higher relative abundance of NO_3_^-^-reducing *R. mucilaginosa* and unclassified sequences, assigned to the health-associated genus *Gemella,* among trained individuals. These health-associated taxa also correlated positively with multiple markers of exercise training status, including V̇O_2max_. Furthermore, NO_3_^-^ and NO_2_^-^ bioavailability was significantly higher in the saliva of trained individuals, as were NO_2_^-^ levels in the plasma. Despite this, no changes were detected in salivary pH or oral NO_2_^-^-production capacity. While our findings suggest differences in the oral microenvironment and tongue dorsum microbiome composition when highly trained competitive athletes are compared to inactive controls, it is unclear if these are direct effects of exercise training or result from other host behaviours.

### Salivary and plasma NO_3_^-^ and NO_2_^-^ are higher in highly trained competitive athletes compared to untrained individuals

Plasma NO_2_^-^ was significantly higher in our trained group than in the untrained group. This finding was not unexpected as previous studies associate elevated plasma NO_2_^-^ with improved exercise performance ^68^ and demonstrate that regular aerobic exercise increases NO_3_^-^ and NO_2_^-^ levels ^30^. This is likely due, in part, to elevated NOS expression, which in turn increases endogenous NO synthesis^69,70^.

The higher level of salivary NO_2_^-^ in the trained group aligns with our previous findings of increased NO_2_^-^ after 8 weeks of high-intensity interval training^67^ and with another study reporting a rise after 10 weeks of combined aerobic and resistance exercise training ^50^. The elevated salivary NO_3_^-^ in the trained group can be contrasted with our previous study, where salivary NO_3_^-^ was unchanged following periods of training and detraining in previously sedentary males^67^. The reasons for this are unclear at present but may be explained by the higher volume of regular exercise carried out by the highly trained competitive athletes in this current study, influencing endogenous NOS-mediated NO production ^30^, increasing systemic NO levels. Under some conditions, this NO can be converted to NO_3_^-^ and NO_2_^-^ inside the body (for example, NO can react with oxygenated haemoglobin forming NO_3_^-^) (Hare et al., 2012). Oral bacteria can then metabolise the resulting NOS-derived NO_3_^-^ and NO_2_^-^, which could give a selective advantage to health-associated species like *R. mucilaginosa*, which reduces both NO_3_^-^ and NO_2_^-^^39,71^.

We found the trained and untrained groups consumed similar levels of dietary NO_3_^-^, suggesting that variations in habitual diet do not explain the elevated salivary NO_3_^-^ and NO_2_^-^ levels. However, methodological limitations must be considered, as this measurement was an estimate only and relied on the participants to accurately record food intake. While it was requested that the participants weigh foods to enable precise NO_3_^-^ quantification, most did not prepare all their own meals as part of their habitual diet. This makes an under- or over-estimation of NO_3_^-^ consumption a possibility, which may have impacted on NO_3_^-^ and NO_2_^-^ levels.

No significant differences were detected in the salivary pH or oral NO_2_^-^ production capacity. Salivary pH is sometimes reported to decrease post-exercise^51^, albeit not in all studies^72^ and a positive association has been detected between oral NO_2_^-^ production capacity and V̇O_2peak_^51^. In contrast with the methodology of our study, Thomas et al.,^51^ collected their samples immediately after the completion of a maximal exercise test. During this period, salivary lactate levels are elevated relative to rest ^22^,Oliveira et al*.*, 2015;^51^, which will have decreased the salivary pH. This could also enhance the activity of NO₃⁻-reducing bacteria, including *R. mucilaginosa*, which utilize lactate as a carbon and electron source during NO₃⁻ reduction ^39,73^. Therefore, these species may only display elevated NO_2_^-^ production capacity immediately post-exercise.

While athlete groups are advised to consume a balanced diet rich in high-quality proteins, vitamins and minerals, which are associated with oral health in the general population^74,75^, they are also advised to use carbohydrate-rich substances to support performance, which will impact on the oral environment and may increase dental disease risk^76^. Higher salivary NO_3_^-^ levels and greater oral NO_3_^-^ reduction capacity have previously been associated with reduced dental caries experience^77^, and dietary NO_3_^-^ supplementation demonstrates protective effects against salivary acidification following carbohydrate consumption^78^). These results suggest that an increase in salivary NO_3_^-^, may have protective effects against the development of dental caries and erosion. However, while a between-group difference in salivary NO_3_^-^ was observed between the trained and untrained individuals, this was of a smaller magnitude compared to that observed following a dietary intervention (see^79^, ^80^,) and no difference was observed in oral NO_2_^-^ production capacity. Additionally, pH decreasing effects, such as a decrease in salivary flow or an increase in carbohydrate consumption in athletes, could limit the pH-buffering effects of the increase in salivary NO_3_^-^ associated with exercise. Thus, the impact of the changes to salivary NO_3_^-^ metabolism associated with exercise training on salivary buffering requires further investigation.

### Highly trained competitive athletes have differences in the composition of their microbiome compared to untrained controls

There were no significant differences in the summed relative abundance of NO_2_^-^-producing microbiome (i.e., species that can produce NO_2_^-^ in the presence of NO_3_^-^) or of confirmed NO_3_^-^-reducing species (i.e., species that have been confirmed to reduce NO_3_^-^ by physiological measurements of NO_3_^-^) ^32^ in our study. While higher NO_3_^-^ and NO_2_^-^ bioavailability in saliva was observed in the trained individuals, NO_2_^-^-producing bacteria must still compete for their required substrate (e.g., lactate). This suggests that the successful growth of one species, such as tongue-associated *R. mucilaginosa* may inhibit the growth of others and may explain why no differences were observed when bacteria were analysed collectively. In a previous exploratory study, *Rothia* increased 4 h after NO_3_^-^-rich beetroot supplement intake^78^, indicating that the fluctuations in species of this genus are dynamic and can happen quickly after substrate administration.

Diversity analysis demonstrated a significant difference in the composition of the tongue dorsum microbiome between the trained and untrained groups. Two previous studies demonstrate similar findings in saliva^21,49^. Conversely, in our previous work, the completion of 75 min of high-intensity interval training per week for eight weeks did not change the overall composition of the tongue dorsum biofilm, relative to remaining sedentary^67^. This suggests that high volumes of intensive exercise training, perhaps coupled with other behavioural changes associated with training for competitive sports, are necessary to modulate the overall composition of the oral microbiome. Alternatively, the microbiome composition may have been affected by between-group differences in the BMI ^81,82^,) or diet ^83,84^. However, while the correlation between *R. mucilaginosa* and unclassified *Gemella* species and V̇O_2max_ in our study should not be interpreted as a causal relationship, it may indicate that the relationship between exercise training and the tongue microbiota warrants further investigation in larger study populations.

We found no evidence that highly trained competitive athletes had a higher relative abundance of bacterial species associated with disease within the supragingival plaque when compared to untrained controls. These findings differ from clinical ^18,19,21,85^ and microbiological findings ^21^ of previous studies of elite-level athletes and suggest that training to compete at the sub-elite level does not result in changes to the supragingival plaque microbiome that could influence dental caries risk. However, due to our small study population, it is possible that the interindividual variation was not large enough to be detected, meaning this result should be confirmed in future studies. Additionally, all individuals indicated they habitually brushed their teeth at least twice per day, contrasting with the tongue microbiota, which could have more time to accumulate, being affected by weekly habits. In future studies, individuals could be informed to abstain from oral hygiene for a short period to see if exercise affects the accumulated plaque community.

The relative abundance of two taxa on a species level were found to be higher on the tongue dorsum of the trained group compared to the untrained controls. These were unclassified *Gemella* species and *R mucilaginosa. Gemella* are core members of the health-associated human oral microbiome (Dewhirst., 2010; ^86^). Furthermore, *Gemella* is periodontal health-associated (Feres et al*.*, 2021) and some *Gemella* species are capable of inhibiting the growth of periodontitis-associated bacteria (e.g., *Porphyromonas gingivalis*) in vitro ^87^. While the small sample size precludes definitive conclusions being reached regarding the role of *R. mucilaginosa* as a biomarker for exercise training status, this work does support previous findings suggesting that exercise training is beneficial for the NO_3_^-^-reducing oral microbiome. *R. mucilaginosa* demonstrates effective NO_3_^-^-reducing capacity and has been proposed as a probiotic species to increase NO availability to improve host health ^39^. It is also one of five NO_2_^-^-producing bacteria seen to increase in relative abundance following consistent exercise training^67^. At the genus level, *Rothia* was also found in higher abundance in the saliva of professional rugby players compared to untrained controls^21^. These preliminary findings provide some early indications that chronic exercise training may result in environmental conditions amenable to the growth of *Rothia* species, especially *R. mucilaginosa*, the human *Rothia* species that is most abundant on the tongue (i.e., tongue tropism)^88^. While it is premature to suggest that the abundance of *R. mucilaginosa* can provide a marker of aerobic fitness, it is an intriguing finding that should be further explored. It should also be noted that *R. mucilaginosa* is a commensal species associated with the absence of halitosis when detected on the tongue^89^ and periodontal health when detected in subgingival plaque^90^, meaning an increase in this bacterium is clearly beneficial for the human host.

These findings were accompanied by multiple strong positive correlations between individual bacteria resident on the tongue dorsum, the volume of exercise training carried out per week and aerobic fitness levels. The completion of regular aerobic exercise training, at a variety of intensities, increases V̇O_2max_^91^. Oral conditions change each time an individual exercises, including an increase in salivary lactate levels^22,51,92^. As previously mentioned, some NO_3_^-^-reducing species, including *R. mucilaginosa* are capable of using lactate as an electron donor during NO_2_^-^ generation^39^. While these findings should be interpreted with caution, it is plausible that the combination of increased NO_3_^-^and NO_2_^-^ availability in the saliva of trained individuals, combined with regular spikes in salivary lactate during intensive exercise, grants certain NO_2_^-^-producing bacteria a survival advantage. Associations were also detected between health-associated bacteria and plasma NO_2_^-^. Species positively associated with exercise training included *R. mucilaginosa* and *Streptococcus salivarius*, a species which has previously demonstrated the ability to increase NO_2_- bioavailability when administered as a probiotic^93^. While *R. mucilaginosa* was the only confirmed NO_3_^-^-reducing bacterium that was significantly higher in the trained group, these exploratory findings suggest that regular exercise may also benefit other NO_3_^-^-reducing species under different conditions, which should be tested in future studies.

### Strengths and limitations

This study used long-read PacBio 16 s rRNA sequencing to conduct precise species-level identification of bacteria, providing data of a higher resolution compared to previously published studies^21,49^. Samples were also collected from environmental niches of relevance to oral NO_2_^-^ production and dental disease development, allowing assessment of the impact of long-term exercise training on the microbiome of these sites. Despite this, multiple bacterial species in the sample could not be classified. This may be due to some bacterial species within the same genus having an identical 16S rRNA gene composition, making it impossible to distinguish these representatives using this mythology. Another limitation could lie within reference databases, particularly for novel or rare taxa where full-length sequences are unavailable. Future research should use metagenomic or metatranscriptomic approaches to identify the bacterial composition on a species- and strain-level, as well as bacterial genomic potential and activity. Additionally, future research may benefit from compositional and functional data with additional methods such as qPCR, allowing for absolute quantification of species of interest. Finally, in some cases, sequencing errors may have led to sequences that could not be classified on a species-level.

However, the study design has some limitations which should be noted. The small sample size makes it challenging to draw robust conclusions regarding the impact of exercise on the oral microbiome and environment. High inter-individual variability in physiological NO_3_^-^ and NO_2_^-^ levels, oral NO_2_^-^ production capacity, and the composition of the oral microbiome may have obscured significant findings, which could be detected with a larger sample size. Therefore, all findings should be considered exploratory and require validation in larger, more comprehensive studies.

Recruitment of untrained participants proved challenging. Participant feedback suggested that using a cycle ergometer instead of a treadmill for maximal exercise testing might reduce apprehension and improve recruitment of sedentary individuals. Between-group differences in BMI, a known factor influencing oral microbiome composition^94,95^, may have contributed to the observed variation in the oral microbiome. Additionally, participants followed different lifestyles and, aside from pre-sampling fasting, no dietary restrictions were imposed to avoid disrupting habitual intake or exercise routines. Although no significant between-group differences in dietary nitrate intake or oral hygiene were detected, individual variability in these behaviours may have influenced microbiome composition and NO bioavailability^71,79,83,96^. Menstrual cycle stage, which affects local inflammation and potentially microbial composition^97^, was not controlled due to practical challenges. The observed differences should therefore be confirmed in a larger population where these confounding factors can be accounted for.

The recruitment criteria aimed to minimise differences between groups likely to alter NO bioavailability^24,98–100^. In addition, while the measurement of NO_2_^-^ following the administration of a NO_3_^-^-rich mouthwash enabled estimation of oral NO_3_^-^-reduction capacity, some of the resulting NO_2_^-^ may have been immediately metabolised further by the oral microbiome^32^. Conversely, other pathways, such as NO oxidation may have increased the concentration of NO_2_^-^ measured post-mouthrinse^32^. Future studies should consider examining the interplay of these metabolic pathways, providing a more comprehensive understanding of oral NO_3_^-^ reduction and the resulting impacts of behavioural changes on oral and systemic health. Finally, although participants reported no history of dental disease and inflammation at the gingival margin was assessed, ethical approval precluded a comprehensive periodontal examination. Additionally, radiographic evaluation was not performed. Consequently, undetected periodontal disease or interproximal carious lesions may have been present in some participants. Future studies should include a thorough clinical and radiographic examination of all participants to better control for confounding variables related to dental diseases.

## Conclusion

To our knowledge, this is the first study to examine differences in the supragingival plaque and tongue microbiome between highly trained and untrained individuals at the species level using long-read 16 s rRNA sequencing. While supragingival plaque showed no differences, the tongue dorsum microbiome differed significantly, with higher relative abundances of health-associated unclassified *Gemella* species and *R. mucilaginosa* in highly trained competitive athletes compared to healthy inactive controls. We also demonstrate that consistent intensive exercise training alters the oral microenvironment, with increased salivary NO_3_^-^ and NO_2_^-^ levels compared to untrained controls, which may further support the development of a health-associated oral microbiome. Despite the limitations of this study, including the small sample size and confounding lifestyle and physiological factors, these findings may have future applications in the fields of sports and dental medicine, such as informing future research into oral microbiome profiles as biomarkers of aerobic fitness, exploring exercise-induced microbial modulation as a means of supporting oral health, and guiding the development of targeted probiotics based on beneficial taxa identified in athletic populations.

## Supplementary Information

Supplementary Information 1.

Supplementary Information 2.

## Data Availability

The datasets generated and/or analysed during the current study are available in the Sequence Read Archive, Sequence Read Archive (SRA) BioProject: PRJNA1244385. XX indicates the participant ID and T or UT indicates training status. The physiological, salivary, plasma and exercise data have been uploaded as one file (Supplementary Datasheet 1).

## Code availability

The data that support the findings of this study are available from the corresponding author, MB, upon reasonable request.

## References

[1] Dewhirst, F. E. et al. The human oral microbiome. *J. Bacteriol.*10.1128/JB.00542-10 (2010). 20656903 10.1128/JB.00542-10 PMC2944498

[2] Griffen, A. L. et al. Distinct and complex bacterial profiles in human periodontitis and health revealed by 16S pyrosequencing. *ISME J.* **6**(6), 1176–1185. 10.1038/ismej.2011.191 (2012). 22170420 10.1038/ismej.2011.191 PMC3358035

[3] Mark Welch, J. L., Ramírez-Puebla, S. T. & Borisy, G. G. Oral Microbiome Geography: Micron-Scale Habitat and Niche. *Cell Host Microbe* **28**(2), 160–168. 10.1016/j.chom.2020.07.009 (2020). 32791109 10.1016/j.chom.2020.07.009 PMC7604680

[4] Jones, A. M. et al. Dietary nitrate and nitric oxide metabolism: mouth, circulation, skeletal muscle, and exercise performance. *Med. Sci. Sports Exerc.* **53**(2), 280. 10.1249/MSS.0000000000002470 (2021). 32735111 10.1249/MSS.0000000000002470

[5] Lundberg, J. O., Carlström, M. & Weitzberg, E. Metabolic effects of dietary nitrate in health and disease. *Cell Metab.* **28**(1), 9–22. 10.1016/j.cmet.2018.06.007 (2018). 29972800 10.1016/j.cmet.2018.06.007

[6] Rajasekaran, J. J. et al. Oral microbiome: a review of its impact on oral and systemic health. *Microorganisms* **12**(9), 1797. 10.3390/microorganisms12091797 (2024). 39338471 10.3390/microorganisms12091797 PMC11434369

[7] Sedghi, L. et al. The oral microbiome: Role of key organisms and complex networks in oral health and disease’. *Periodontol.* **87**(1), 107–131. 10.1111/prd.12393 (2000). 10.1111/prd.12393 PMC8457218 34463991

[8] Rosier, B. T., Marsh, P. D. & Mira, A. Resilience of the oral microbiota in health: mechanisms that prevent dysbiosis. *J. Dent. Res.*10.1177/0022034517742139 (2018). 29195050 10.1177/0022034517742139

[9] Peng, X. et al. Oral microbiota in human systematic diseases. *Int. J. Oral Sci.* **14**(1), 1–11. 10.1038/s41368-022-00163-7 (2022). 35236828 10.1038/s41368-022-00163-7 PMC8891310

[10] Medapati, A. R. & Pachava, S. Effect of physical activity on oral health: a systematic review. *J. In. Assoc. Public Health Dentistry* **20**(2), 125. 10.4103/jiaphd.jiaphd_142_21 (2022).

[11] Baskaradoss, J. K. et al. Association between frequency of toothbrushing and metabolic syndrome among adolescents: A 5-year follow-up study. *Int. J. Environ. Res. Public Health* **19**(1), 508. 10.3390/ijerph19010508 (2022). 35010768 10.3390/ijerph19010508 PMC8744688

[12] Virtanen, J. I. et al. Physical activity, bmi and oral health behaviour among adolescents: finnish school health promotion study. *Eur. J. Pub. Health* **29**(2), 296–302. 10.1093/eurpub/cky193 (2019). 30252075 10.1093/eurpub/cky193

[13] de Ferreira, R. et al. Physical activity reduces the prevalence of periodontal disease: systematic review and meta-analysis. *Front. Physiol.*10.3389/fphys.2019.00234 (2019). 30949062 10.3389/fphys.2019.00234 PMC6438044

[14] Huttunen, M. et al. The association between dental caries and physical activity, physical fitness, and background factors among Finnish male conscripts. *Odontology* **111**(1), 192–200. 10.1007/s10266-022-00717-5 (2023). 35612763 10.1007/s10266-022-00717-5 PMC9810556

[15] Inui, A. et al. Teeth and physical fitness in a community-dwelling 40 to 79-year-old Japanese population. *Clin. Interv. Aging* **11**, 873–878. 10.2147/CIA.S108498 (2016). 27418813 10.2147/CIA.S108498 PMC4933564

[16] Popa, P. Ș et al. Study on the influence of regular physical activity on children’s oral health. *Children* **10**(6), 946. 10.3390/children10060946 (2023). 37371181 10.3390/children10060946 PMC10296963

[17] Needleman, I. et al. Oral health and impact on performance of athletes participating in the London 2012 Olympic Games: a cross-sectional study - PMC. *Br J Sports Med* **47**(16), 1054–1058 (2013). 24068332 10.1136/bjsports-2013-092891 PMC3812828

[18] Gallagher, J. et al. Oral health and performance impacts in elite and professional athletes. *Commun. Dent. Oral Epidemiol.* **46**(6), 563–568. 10.1111/cdoe.12392 (2018). 10.1111/cdoe.12392 29938820

[19] Kragt, L. et al. Oral health among Dutch elite athletes prior to Rio 2016. *Phys. Sportsmed.* **47**(2), 182–188. 10.1080/00913847.2018.1546105 (2019). 30408425 10.1080/00913847.2018.1546105

[20] Gallagher, J. et al. Oral health-related behaviours reported by elite and professional athletes. *Br. Dent. J.* **227**(4), 276–280. 10.1038/s41415-019-0617-8 (2019). 31444443 10.1038/s41415-019-0617-8

[21] Minty, M. et al. Oral health and microbiota status in professional rugby players: A case-control study. *J Dent* **79**, 53–60. 10.1016/j.jdent.2018.10.001 (2018). 30292825 10.1016/j.jdent.2018.10.001

[22] Santos, R. V. T. et al. Effects of a 30-km race upon salivary lactate correlation with blood lactate. *Comp. Biochem. Physiol. B: Biochem. Mol. Biol.* **145**(1), 114–117. 10.1016/j.cbpb.2006.07.001 (2006). 16893666 10.1016/j.cbpb.2006.07.001

[23] Armstrong, L. E. Rehydration during endurance exercise: challenges, research, options, methods. *Nutrients* **13**(3), 887. 10.3390/nu13030887 (2021). 33803421 10.3390/nu13030887 PMC8001428

[24] Bescos, R. et al. Modulation of oral microbiota: A new frontier in exercise supplementation. *PharmaNutrition* **14**, 100230. 10.1016/j.phanu.2020.100230 (2020).

[25] Burleigh, M. et al. Nitrate-rich beetroot juice offsets salivary acidity following carbohydrate ingestion before and after endurance exercise in healthy male runners. *PLoS ONE* **15**(12), e0243755 (2020). 33320868 10.1371/journal.pone.0243755 PMC7737958

[26] Gibala, M. J., Bostad, W. & McCarthy, D. G. Physiological adaptations to interval training to promote endurance. *Curr. Opin. Physio.* **10**, 180–184. 10.1016/j.cophys.2019.05.013 (2019).

[27] Mishica, C. et al. Performance-related physiological changes induced by one year of endurance training in young athletes. *Front. Sports Active Living*10.3389/fspor.2023.1149968 (2023). 10.3389/fspor.2023.1149968 PMC10206302 37234748

[28] Green, D. J. et al. Effect of exercise training on endothelium-derived nitric oxide function in humans. *J. Physiol.* **561**(Pt 1), 1–25. 10.1113/jphysiol.2004.068197 (2004). 15375191 10.1113/jphysiol.2004.068197 PMC1665322

[29] Su, S.-H., Jen, C. J. & Chen, H. NO signaling in exercise training-induced anti-apoptotic effects in human neutrophils. *Biochem. Biophys. Res. Commun.* **405**(1), 58–63. 10.1016/j.bbrc.2010.12.123 (2011). 21195695 10.1016/j.bbrc.2010.12.123

[30] Arefirad, T. et al. Effect of exercise training on nitric oxide and nitrate/nitrite (NOx) production: A systematic review and meta-analysis. *Front. Physiol.*10.3389/fphys.2022.953912 (2022). 36267589 10.3389/fphys.2022.953912 PMC9576949

[31] Hezel, M. P. & Weitzberg, E. The oral microbiome and nitric oxide homoeostasis. *Oral Dis.* **21**(1), 7–16. 10.1111/odi.12157 (2015). 23837897 10.1111/odi.12157

[32] Rosier, B. T. et al. The importance of nitrate reduction for oral health. *J Dent Res.* **101**(8), 887–897. 10.1177/00220345221080982 (2022). 35196931 10.1177/00220345221080982

[33] Gao, C. et al. The effects of dietary nitrate supplementation on endurance exercise performance and cardiorespiratory measures in healthy adults: a systematic review and meta-analysis. *J. Int. Soc. Sports Nutr.* **18**(1), 55. 10.1186/s12970-021-00450-4 (2021). 34243756 10.1186/s12970-021-00450-4 PMC8268374

[34] Coggan, A. R. et al. Effect of dietary nitrate on human muscle power: a systematic review and individual participant data meta-analysis. *J. Int. Soc. Sports Nutr.* **18**(1), 66. 10.1186/s12970-021-00463-z (2021). 34625064 10.1186/s12970-021-00463-z PMC8501726

[35] Van De Walle, G. P. & Vukovich, M. D. The effect of nitrate supplementation on exercise tolerance and performance: a systematic review and meta-analysis. *J. Strength Cond. Res.* **32**(6), 1796–1808. 10.1519/JSC.0000000000002046 (2018). 29786633 10.1519/JSC.0000000000002046

[36] Liu, H. et al. From nitrate to NO: potential effects of nitrate-reducing bacteria on systemic health and disease. *Eur. J. Med. Res.* **28**, 425. 10.1186/s40001-023-01413-y (2023). 37821966 10.1186/s40001-023-01413-y PMC10566198

[37] Lundberg, J. O., Weitzberg, E. & Gladwin, M. T. The nitrate-nitrite-nitric oxide pathway in physiology and therapeutics. *Nat. Rev. Drug Discovery* **7**(2), 156–167. 10.1038/nrd2466 (2008). 18167491 10.1038/nrd2466

[38] Qin, L. et al. Sialin (SLC17A5) functions as a nitrate transporter in the plasma membrane. *Proc. Natl. Acad. Sci. U S A.* **109**(33), 13434–13439. 10.1073/pnas.1116633109 (2012). 22778404 10.1073/pnas.1116633109 PMC3421170

[39] Rosier, B. T. et al. Isolation and characterization of nitrate-reducing bacteria as potential probiotics for oral and systemic health. *Front. Microbiol.* **11**, 555465. 10.3389/fmicb.2020.555465 (2020). 33042063 10.3389/fmicb.2020.555465 PMC7522554

[40] Aneman, A. et al. Continuous measurement of gastric nitric oxide production. *Am. J. Physiol.* **271**(6 Pt 1), G1039-1042. 10.1152/ajpgi.1996.271.6.G1039 (1996). 8997248 10.1152/ajpgi.1996.271.6.G1039

[41] Bryan, N. S. et al. Dietary nitrite supplementation protects against myocardial ischemia-reperfusion injury. *Proc. Natl. Acad. Sci.* **104**(48), 19144–19149. 10.1073/pnas.0706579104 (2007). 18025468 10.1073/pnas.0706579104 PMC2141922

[42] Carlström, M. et al. Dietary nitrate attenuates oxidative stress, prevents cardiac and renal injuries, and reduces blood pressure in salt-induced hypertension. *Cardiovasc. Res.* **89**(3), 574–585. 10.1093/cvr/cvq366 (2011). 21097806 10.1093/cvr/cvq366

[43] Cosby, K. et al. Nitrite reduction to nitric oxide by deoxyhemoglobin vasodilates the human circulation. *Nat. Med.* **9**(12), 1498–1505. 10.1038/nm954 (2003). 14595407 10.1038/nm954

[44] Li, H. et al. Nitric oxide production from nitrite occurs primarily in tissues not in the blood: critical role of xanthine oxidase and aldehyde oxidase. *J. Biol. Chem.* **283**(26), 17855–17863. 10.1074/jbc.M801785200 (2008). 18424432 10.1074/jbc.M801785200 PMC2440597

[45] Webb, A. J. et al. Acute blood pressure lowering, vasoprotective, and antiplatelet properties of dietary nitrate via bioconversion to nitrite. *Hypertension* **51**(3), 784–790. 10.1161/HYPERTENSIONAHA.107.103523 (2008). 18250365 10.1161/HYPERTENSIONAHA.107.103523 PMC2839282

[46] Gross, E. L. et al. Beyond streptococcus mutans: dental caries onset linked to multiple species by 16S rRNA community analysis. *PLoS ONE* **7**(10), e47722. 10.1371/journal.pone.0047722 (2012). 23091642 10.1371/journal.pone.0047722 PMC3472979

[47] Korona-Glowniak, I. et al. Streptococcus sobrinus as a predominant oral bacteria related to the occurrence of dental caries in polish children at 12 years old. *Int. J. Environ. Res. Public Health* **19**(22), 15005. 10.3390/ijerph192215005 (2022). 36429724 10.3390/ijerph192215005 PMC9690266

[48] Oehmcke, S. et al. Streptococcal M proteins and their role as virulence determinants’, *Clinica**Chimica**Acta*. *Int. J. Clin. Chem.* **411**(17–18), 1172–1180. 10.1016/j.cca.2010.04.032 (2010). 10.1016/j.cca.2010.04.032 20452338

[49] Kalabiska, I. et al. The oral microbiome profile of water polo players aged 16–20. *Sports (Basel, Switzerland)* **11**(11), 216. 10.3390/sports11110216 (2023). 37999433 10.3390/sports11110216 PMC10674641

[50] Amaral, A. L. et al. Effects of combined exercise on salivary oxidative stress in hypertensive and normotensive postmenopausal women. *Motriz Revista Educação Física*10.1590/S1980-657420220012321 (2021).

[51] Thomas, B. et al. The oral nitrate-reducing capacity correlates with peak power output and peak oxygen uptake in healthy humans. *Nitric Oxide Biol. Chem.* **87**, 43–51. 10.1016/j.niox.2019.03.001 (2019). 10.1016/j.niox.2019.03.001 30853629

[52] Shaw, L. et al. The human salivary microbiome is shaped by shared environment rather than genetics: evidence from a large family of closely related individuals. *MBio* **8**(5), e01237-e1317. 10.1128/mBio.01237-17 (2017). 28900019 10.1128/mBio.01237-17 PMC5596345

[53] Callahan, B. J., McMurdie, P. J. & Holmes, S. P. Exact sequence variants should replace operational taxonomic units in marker-gene data analysis. *ISME J.* **11**(12), 2639–2643. 10.1038/ismej.2017.119 (2017). 28731476 10.1038/ismej.2017.119 PMC5702726

[54] McKay, A. K. A. et al. Defining training and performance caliber: a participant classification framework. *Int. J. Sports Physiol. Perform.* **17**(2), 317–331. 10.1123/ijspp.2021-0451 (2021). 10.1123/ijspp.2021-0451 34965513

[55] Navazesh, M. & Kumar, S. K. S. (2008) ‘Measuring salivary flow: challenges and opportunities’. *J. Am. Dental Assoc.* **139**, 35S-40S. 10.14219/jada.archive.2008.0353 (1939). 10.14219/jada.archive.2008.0353 18460678

[56] R Core Team (2016) *R Core Team (2016) R A Language and Environment for Statistical Computing. R Foundation for Statistical Computing, Vienna, Austria. - References - Scientific Research Publishing*. https://www.scirp.org/reference/ReferencesPapers?ReferenceID=2010931 (Accessed: 11 January 2024).

[57] Callahan, B. J. et al. DADA2: High-resolution sample inference from Illumina amplicon data. *Nat. Methods* **13**(7), 581–583. 10.1038/nmeth.3869 (2016). 27214047 10.1038/nmeth.3869 PMC4927377

[58] McLaren, M. and Callahan, B. (2021) ‘Silva 138.1 Prokaryotic SSU Taxonomic Training Data Formatted for DADA2 [Data Set] (Zenodo)’. 10.5281/zenodo.4587955.

[59] Quast, C. et al. The SILVA ribosomal RNA gene database project: improved data processing and web-based tools. *Nucleic Acids Res.* **41**(D1), D590–D596. 10.1093/nar/gks1219 (2013). 23193283 10.1093/nar/gks1219 PMC3531112

[60] Yilmaz, P. et al. The SILVA and “All-species living tree project (LTP)” taxonomic frameworks. *Nucleic Acids Res.* **42**(D1), D643–D648. 10.1093/nar/gkt1209 (2014). 24293649 10.1093/nar/gkt1209 PMC3965112

[61] Oksanen, J. *et al.* (2022) ‘Vegan: Community Ecology Package. R package’.

[62] Lin, H. & Peddada, S. D. Analysis of compositions of microbiomes with bias correction. *Nat. Commun.* **11**(1), 3514. 10.1038/s41467-020-17041-7 (2020). 32665548 10.1038/s41467-020-17041-7 PMC7360769

[63] González, I. et al. Visualising associations between paired “omics” data sets. *BioData Mining* **5**(1), 19. 10.1186/1756-0381-5-19 (2012). 23148523 10.1186/1756-0381-5-19 PMC3630015

[64] Le Cao, K.-A. *et al.* (2016) ‘mixOmics: Omics Data Integration Project. R package’. https://CRAN.R-project.org/package=mixOmics.

[65] Johnston, W. et al. Mechanical biofilm disruption causes microbial and immunological shifts in periodontitis patients. *Sci Rep.* **11**(1), 9796. 10.1038/s41598-021-89002-z (2021). 33963212 10.1038/s41598-021-89002-z PMC8105330

[66] Rohart, F. et al. mixOmics: An R package for ’omics feature selection and multiple data integration. *PLoS Comput. Biol.* **13**(11), e1005752. 10.1371/journal.pcbi.1005752 (2017). 29099853 10.1371/journal.pcbi.1005752 PMC5687754

[67] Simpson, A. et al. Eight weeks of high-intensity interval training alters the tongue microbiome and impacts nitrate and nitrite levels in previously sedentary men. *Free Radical Biol. Med.* **231**, 11–22. 10.1016/j.freeradbiomed.2025.02.006 (2025). 39923866 10.1016/j.freeradbiomed.2025.02.006

[68] Rassaf, T. et al. Nitric oxide synthase-derived plasma nitrite predicts exercise capacity. *Br. J. Sports Med.* **41**(10), 669–673. 10.1136/bjsm.2007.035758 (2007). 17496072 10.1136/bjsm.2007.035758 PMC2465183

[69] McConell, G. K. et al. Skeletal muscle nNOSμ protein content is increased by exercise training in humans. *Am. J. Physiol. Regulatory, Integrative Comparative Physiol.* **293**(2), R821–R828. 10.1152/ajpregu.00796.2006 (2007). 10.1152/ajpregu.00796.2006 17459909

[70] Rudnick, J. et al. Differential expression of nitric oxide synthases (NOS 1–3) in human skeletal muscle following exercise countermeasure during 12 weeks of bed rest. *FASEB J. Official Publi. Federation Am. Soc. Exp. Biol.* **18**(11), 1228–1230. 10.1096/fj.03-0792fje (2004). 10.1096/fj.03-0792fje 15180967

[71] Moran, S. P. et al. The effects of nitrate on the oral microbiome: a systematic review investigating prebiotic potential. *J. Oral Microbiol.*10.1080/20002297.2024.2322228 (2024). 38420038 10.1080/20002297.2024.2322228 PMC10901185

[72] Ligtenberg, A. J. M. et al. The effect of physical exercise on salivary secretion of MUC5B, amylase and lysozyme. *Arch. Oral Biol.* **60**(11), 1639–1644. 10.1016/j.archoralbio.2015.07.012 (2015). 26351746 10.1016/j.archoralbio.2015.07.012

[73] Hoshino, E. & Araya, A. Lactate degradation by polysaccharide-producing Neisseria isolated from human dental plaque. *Arch. Oral Biol.* **25**(3), 211–212. 10.1016/0003-9969(80)90023-0 (1980). 6930960 10.1016/0003-9969(80)90023-0

[74] Gondivkar, S. M. et al. Nutrition and oral health. *Dis. Mon.* **65**(6), 147–154. 10.1016/j.disamonth.2018.09.009 (2019). 30293649 10.1016/j.disamonth.2018.09.009

[75] Najeeb, S. et al. The Role of Nutrition in Periodontal Health: An Update. *Nutrients* **8**(9), 530. 10.3390/nu8090530 (2016). 27589794 10.3390/nu8090530 PMC5037517

[76] Valenzuela, M. J. et al. Effect of sugar-sweetened beverages on oral health: a systematic review and meta-analysis. *Eur. J. Pub. Health* **31**(1), 122–129 (2021). 32830237 10.1093/eurpub/ckaa147

[77] Doel, J. J. et al. Protective effect of salivary nitrate and microbial nitrate reductase activity against caries. *Eur. J. Oral Sci.* **112**(5), 424–428. 10.1111/j.1600-0722.2004.00153.x (2004). 15458501 10.1111/j.1600-0722.2004.00153.x

[78] Rosier, B. T. et al. A single dose of nitrate increases resilience against acidification derived from sugar fermentation by the oral microbiome. *Front. Cell Infect Microbiol.* **11**, 692883. 10.3389/fcimb.2021.692883 (2021). 34195102 10.3389/fcimb.2021.692883 PMC8238012

[79] Burleigh, M. C. et al. Salivary nitrite production is elevated in individuals with a higher abundance of oral nitrate-reducing bacteria. *Free Radic. Biol. Med.* **120**, 80–88. 10.1016/j.freeradbiomed.2018.03.023 (2018). 29550328 10.1016/j.freeradbiomed.2018.03.023

[80] Vanhatalo, A. et al. Nitrate-responsive oral microbiome modulates nitric oxide homeostasis and blood pressure in humans. *Free Radic. Biol. Med.* **124**, 21–30. 10.1016/j.freeradbiomed.2018.05.078 (2018). 29807159 10.1016/j.freeradbiomed.2018.05.078 PMC6191927

[81] Wu, Y. et al. Characterization of the salivary microbiome in people with obesity. *PeerJ* **6**, e4458. 10.7717/peerj.4458 (2018). 29576948 10.7717/peerj.4458 PMC5858547

[82] Ma, T. et al. Characterization of the oral and gut microbiome in children with obesity aged 3 to 5 years. *Front. Cellular Infect. Microbiol.*10.3389/fcimb.2023.1102650 (2023). 10.3389/fcimb.2023.1102650 PMC10090557 37065198

[83] Angarita-Díaz, M. D. P. et al. Does high sugar intake really alter the oral microbiota?: A systematic review. *Clin. Exp. Dental Res.* **8**(6), 1376–1390. 10.1002/cre2.640 (2022). 10.1002/cre2.640 PMC9760141 35946056

[84] Cato, L. E. et al. Low Carbohydrate, High Fat Diet Alters the Oral Microbiome without Negating the Nitrite Response to Beetroot Juice Supplementation. *Nutrients* **15**(24), 5123. 10.3390/nu15245123 (2023). 38140382 10.3390/nu15245123 PMC10745889

[85] Needleman, I. et al. Poor oral health including active caries in 187 UK professional male football players: clinical dental examination performed by dentists. *Br J Sports Med* **50**(1), 41–44. 10.1136/bjsports-2015-094953 (2016). 26527674 10.1136/bjsports-2015-094953

[86] Torres-Morales, J. et al. Site-specialization of human oral Gemella species. *J. Oral Microbiol.* **15**(1), 2225261. 10.1080/20002297.2023.2225261 (2023). 37361319 10.1080/20002297.2023.2225261 PMC10288933

[87] Miyoshi, T. et al. Gemella haemolysans inhibits the growth of the periodontal pathogen Porphyromonas gingivalis. *Sci. Rep.* **11**(1), 11742. 10.1038/s41598-021-91267-3 (2021). 34083694 10.1038/s41598-021-91267-3 PMC8175725

[88] Wilbert, S. A., Welch, M. J. L. & Borisy, G. G. Spatial ecology of the human tongue dorsum microbiome. *Cell Rep* **30**(12), 4003-4015.e3. 10.1016/j.celrep.2020.02.097 (2020). 32209464 10.1016/j.celrep.2020.02.097 PMC7179516

[89] Carda-Diéguez, M. et al. The tongue biofilm metatranscriptome identifies metabolic pathways associated with the presence or absence of halitosis. *npj Biofilms and Microb.* **8**(1), 1–10. 10.1038/s41522-022-00364-2 (2022). 10.1038/s41522-022-00364-2 PMC9763428 36535943

[90] Feres, M. et al. ‘Did Omics change periodontal therapy?’. *Periodontol.* **85**(1), 182–209. 10.1111/prd.12358 (2000). 10.1111/prd.12358 33226695

[91] Scribbans, T. D. et al. The effect of training intensity on VO2max in young healthy adults: a meta-regression and meta-analysis. *Int. J. Exercise Sci.* **9**(2), 230–247 (2016). 10.70252/HHBR9374 PMC4836566 27182424

[92] dos Oliveira, L. et al. Salivary and blood lactate kinetics in response to maximal workload on cycle ergometer. *Revista Brasileira de Cineantropometria & Desempenho Humano* **17**, 565–574. 10.5007/1980-0037.2015v17n5p565 (2015).

[93] Burleigh, M. C. et al. The probiotic streptococcus salivarius m18 increases plasma nitrite but does not alter blood pressure: a pilot randomised controlled trial. *Appl. Microbiol.* **3**(3), 774–785. 10.3390/applmicrobiol3030054 (2023).

[94] Rahman, B. et al. Dysbiosis of the subgingival microbiome and relation to periodontal disease in association with obesity and overweight. *Nutrients* **15**(4), 826. 10.3390/nu15040826 (2023). 36839184 10.3390/nu15040826 PMC9965236

[95] Stefura, T. et al. Differences in compositions of oral and fecal microbiota between patients with obesity and controls. *Medicina* **57**(7), 678. 10.3390/medicina57070678 (2021). 34209298 10.3390/medicina57070678 PMC8306358

[96] Avoort, C. M. T. et al. A nitrate-rich vegetable intervention elevates plasma nitrate and nitrite concentrations and reduces blood pressure in healthy young adults. *J. Acad. Nutr. Diet.* **120**(8), 1305–1317. 10.1016/j.jand.2020.02.014 (2020). 32386891 10.1016/j.jand.2020.02.014

[97] Asaad, N. K. & Abbood, H. M. Comparing gingival inflammation and salivary acidity to hormonal variation during menstruation. *Saudi Dental J.* **35**(3), 251. 10.1016/j.sdentj.2023.02.001 (2023). 10.1016/j.sdentj.2023.02.001 PMC10114131 37091273

[98] Bailey, S. J. et al. Improvement in blood pressure after short-term inorganic nitrate supplementation is attenuated in cigarette smokers compared to non-smoking controls. *Nitric Oxide Biol. Chem.* **61**, 29–37. 10.1016/j.niox.2016.10.002 (2016). 10.1016/j.niox.2016.10.002 27744007

[99] Brookes, Z. L. S. et al. Effects of chlorhexidine mouthwash on the oral microbiome. *J Dent.* **113**, 103768. 10.1016/j.jdent.2021.103768 (2020). 10.1016/j.jdent.2021.103768 34418463

[100] Lundberg, J. O. & Weitzberg, E. Nitric oxide signaling in health and disease. *Cell* **185**(16), 2853–2878. 10.1016/j.cell.2022.06.010 (2022). 35931019 10.1016/j.cell.2022.06.010

