# Supplementary material for: A comparison of oral microbiome composition between highly trained competitive athletes and untrained controls
Source: Sci Rep. 2025 Aug 28;15:31736. doi: 10.1038/s41598-025-16835-3 (PMC12394404; doi:10.1038/s41598-025-16835-3)
Supplement: Supplementary file 2 — Supplementary Information 2. [file 41598_2025_16835_MOESM2_ESM.docx]

| Supplementary Table 1: Nitrite-Producing Species (from Rosier et al., 2022) |
| --- |
| Species  *Actinomyces georgiae, Actinomyces graevenitzii, Actinomyces hongkongensis, Actinomyces johnsonii, Actinomyces lingnae, Actinomyces massiliensis, Actinomyces naeslundii, Actinomyces odontolyticus, Actinomyces oris, Actinomyces viscosus, Capnocytophaga gingivalis, Capnocytophaga ochracea, Capnocytophaga sputigena, Corynebacterium durum, Corynebacterium matruchotii, Cutibacterium acnes, Eikenella corrodens*  *Fusobacterium nucleatum, Granulicatella adiacens, Haemophilus parainfluenzae, Haemophilus segnis, Kingella denitrificans, Neisseria elongate, Neisseria flavescens, Neisseria macacae, Neisseria mucosa, Neisseria oralis, Neisseria sicca, Neisseria subflava. Paraburkholderia fungorum, Prevotella melanginogenica, Propionibacterium acnes, Pseudopropionibacterium propionicum, Rothia aeria, Rothia dentocariosa, Rothia mucilaginosa, Schaalia odontolytica, Selenomonas artemidis, Selenomonas flueggei, Selenomonas noxia, Streptococcus australis, Streptococcus infantis, Streptococcus mitis, Streptococcus mutans, Streptococcus oralis, Streptococcus parasanguinis, Streptococcus salivarius, Streptococcus sanguinis, Veillonella atypica, Veillonella dispar, Veillonella parvula, Veillonella tobetsuensis* |

| Supplementary Table 2: Nitrate-Reducing Species (from Rosier et al., 2022) |
| --- |
| Species  *Actinomyces georgiae, Actinomyces graevenitzii, Actinomyces hongkongensis, Actinomyces johnsonii, Actinomyces lingnae, Actinomyces massiliensis, Actinomyces naeslundii, Actinomyces odontolyticus, Actinomyces oris, Actinomyces viscosus, Cutibacterium acnes, Kingella denitrificans, Neisseria elongate, Neisseria flavescens, Neisseria macacae, Neisseria mucosa, Neisseria oralis, Neisseria sicca,*  *Neisseria subflava, Propionibacterium acnes, Pseudopropionibacterium propionicum, Rothia aeria, Rothia dentocariosa, Rothia mucilaginosa, Schaalia odontolytica, Veillonella atypica, Veillonella dispar, Veillonella parvula, Veillonella tobetsuensis* |

**Supplementary Figure 1**


Supplementary Figure 1. Assessing the impact of long-term intensive aerobic exercise training on the diversity and composition of the oral microbiome at the genus level. (a) Rarefaction curves for observed genera. Differences in alpha diversity indices (b) dbp. (c) Shannon index. (d) Chao1 index between trained and untrained participants^⊗^ and between the microbiome of the tongue and subgingival plaque in participants of the same training status^∩^. Data shown as median with IQR. Differences in beta-diversity between the microbiome of trained and untrained participants (e) Tongue dorsum. (f) Subgingival plaque. (g) Combined differences in beta-diversity between microbiome samples of different groups. Most abundant genera in the microbiome of trained and untrained participants (h) Tongue dorsum. (i) Supragingival plaque in the trained and untrained groups. All genera with relative abundance >1% are shown, with the remaining species described as ‘other’. ^⊗^Wilcoxon test ^∩^.Wilcoxon signed-rank test. * *p*<0.05, ** *p*<0.01.

**Supplementary Figure 2**

Supplementary Figure 2. Assessing differences in the relative abundance of all known NO_2_^-^ -producing species (a) and all known NO_3_^-^ -reducing species (b) on the tongue dorsum and in the supragingival plaque between the trained (TR) and untrained (UTR) participants. Data shown as median with IQR. Each point represents a participant in the study. Groups compared using a Wilcoxon test, carried out on the ANCOMBC-2 transformed relative abundance values. No significant differences were detected.

**Supplementary Figure 3**

**
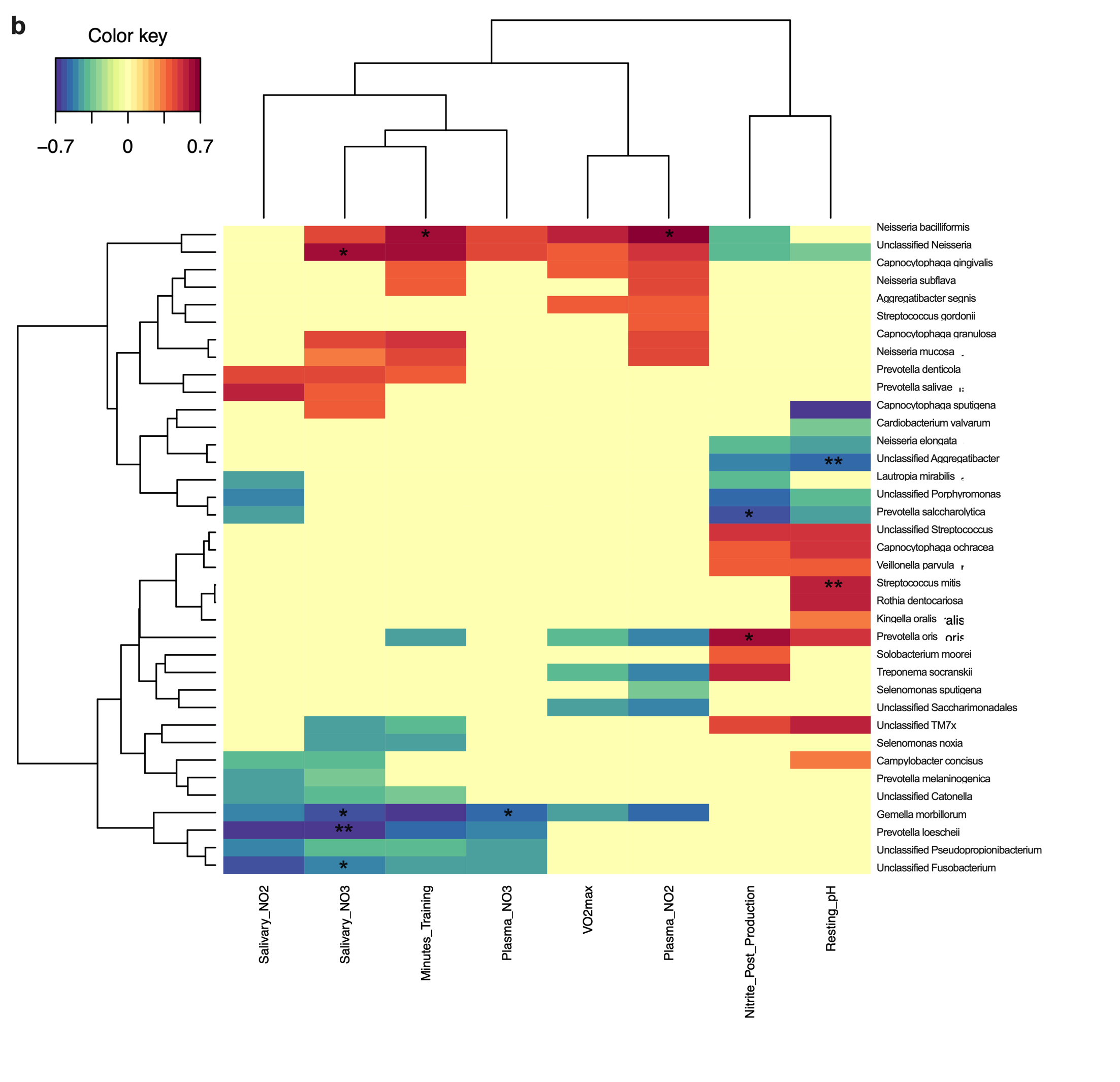
**

Supplementary Figure 3 - Shows associations between the composition of the dental plaque microbiome, NO_3_^-^ and NO_2_^-^ levels and the aerobic training status of all participants. Heatmaps with associations between markers of exercise training status, NO_3_^-^ and NO_2_^-^ levels, salivary pH and oral NO_2_^-^ production levels and bacterial abundance at (a) the genus level and (b) species level. These heatmaps show the association between bacterial abundances after ANCOM-BC transformation and other parameters, which were obtained based on their projection onto a correlation circle plot derived from a principal component analysis. Only negative associations below -0.4 and positive associations above 0.4 between species are shown. To complement the association heatmaps, correlations between species and other parameters were determined with Spearman's rho and marked with asterisks on the heatmap (* *p*-unadjusted <0.05, ** *p*-unadjusted <0.01).

**Supplementary Figure 4**

Supplementary Figure 4: Correlation circle plots for association networks. Association between genera and species are obtained based on their projection onto a correlation circle plot derived from a principal component analysis, as described by González *et al.*, 2012). This was performed at genus-level for the microbiome of the tongue dorsum (a) and supragingival plaque (b), and species-level level for the microbiome of the tongue dorsum (c) and supragingival plaque (d). Associations were confirmed with Spearman-Rho correlation coefficients, see Supplementary File 2.
